# *Mycobacterium abscessus* infection leads to enhanced production of type 1 interferon and NLRP3 inflammasome activation in murine macrophages via mitochondrial oxidative stress

**DOI:** 10.1371/journal.ppat.1008294

**Published:** 2020-03-25

**Authors:** Bo-Ram Kim, Byoung-Jun Kim, Yoon-Hoh Kook, Bum-Joon Kim

**Affiliations:** Department of Microbiology and Immunology, Biomedical Sciences, Liver Research Institute and Cancer Research Institute, College of Medicine, Seoul National University, Seoul, Korea; University of Washington, UNITED STATES

## Abstract

*Mycobacterium abscessus* (MAB) is a rapidly growing mycobacterium (RGM), and infections with this pathogen have been increasing worldwide. Recently, we reported that rough type (MAB-R) but not smooth type (MAB-S) strains enhanced type 1 interferon (IFN-I) secretion via bacterial phagosome escape, contributing to increased virulence. Here, we sought to investigate the role of mitochondrial oxidative stress in bacterial survival, IFN-I secretion and NLRP3 inflammasome activation in MAB-infected murine macrophages. We found that live but not heat-killed (HK) MAB-R strains increased mitochondrial ROS (mtROS) and increased release of oxidized mitochondrial DNA (mtDNA) into the cytosol of murine macrophages compared to the effects of live MAB-S strains, resulting in enhanced NLRP3 inflammasome-mediated IL-1β and cGAS-STING-dependent IFN-I production. Treatment of the infected macrophages with mtROS-modulating agents such as mito-TEMPO or cyclosporin A reduced cytosolic oxidized mtDNA, which inhibited the MAB-R strain-induced production of IL-1β and IFN-I. The reduced cytosolic oxidized mtDNA also inhibited intracellular growth of MAB-R strains via cytosolic escape following phagosomal rupture and via IFN-I-mediated cell-to-cell spreading. Moreover, our data showed that mtROS-dependent IFN-I production inhibited IL-1β production, further contributing to MAB-R intracellular survival in murine macrophages. In conclusion, our data indicated that MAB-R strains enhanced IFN-I and IL-1β production by inducing mtROS as a pathogen-associated molecular pattern (PAMP). These events also enhance bacterial survival in macrophages and dampen inflammation, which contribute to the pathogenesis of MAB-R strains.

## Author summary

MAB infections have gained increasing attention due to their clinical significance. Mitochondrial oxidative stress regulates intrinsic innate immune responses mainly via IFN-I or IL-1β production, which affects the pathogenesis of several pathogens, including *Mycobacterium tuberculosis* infections. Here, we found that virulent MAB-R but not MAB-S strains induced mtROS in infected macrophages, resulting in enhanced IFN-I and IL-1β production by the release of oxidized mtDNA into the cytosol. Furthermore, increased mtROS exerted a pro-bacterial effect by inducing IFN-I-mediated escape of MAB-R into the cytosol. In MAB-R-infected murine macrophages, mtROS-induced IFN-I also inhibited IL-1β production exerting an antibacterial effect, further contributing to intracellular bacterial survival. Our data indicate that mtROS play an important role in MAB-R pathogenesis by facilitating bacterial survival and dampening inflammation in macrophages as a kind of specific class of PAMP. mtROS may be a valuable target for the treatment of virulent MAB infections.

## Introduction

*Mycobacterium abscessus* (MAB) is a species of rapidly growing mycobacteria (RGM) that leads to pulmonary infection [1–4], and it has gained increasing attention worldwide as a common pathogen in lung diseases, especially in cystic fibrosis patients [5–7]. MAB strains lead to nosocomial infections [8, 9], and MAB infections are well known for being difficult to treat [10, 11], due to both natural broad-spectrum and acquired resistance, with disparate patterns of antibiotic susceptibility observed between different clinical strains [12–16].

MAB consists of two colony morphotypes, one with rough colonies (MAB-R) that lack glycopeptidolipid (GPL) in the outer membrane and the other with smooth colonies (MAB-S) that produce GPL [17–19]. It has been reported that there are some distinct phenotypes between the two types. In general, MAB-R causes a more severe inflammatory response in infected macrophages or in infected mice compared to the effects of MAB-S strains [18, 20, 21]. However, MAB-S has an advantage in survival in the environment due to GPL-based biofilm formation, which also contributes to its enhanced resistance to bacterial apoptotic cell death [2, 20, 21].

Recently, we reported that, similar to *M*. *tuberculosis* (*Mtb*), the distinct virulence potential between MAB-R and -S strains also depends on their capacity to induce type 1 interferon (IFN-I) production [22]. In this study, we showed that although there is no orthologue of *Mtb* ESX-1 in the MAB genome, MAB-R but not MAB-S strains also induce IFN-I production via escape of the bacteria into the cytosol after phagosomal rupture, which in turn results in enhanced bacterial survival in infected macrophages via IFN-I-dependent cell-to-cell spreading. This highlights the significance of IFN-I production in the pathogenesis of MAB infections.

In the *Mtb* infection model, it has been previously reported that bacteria that escape into the cytosol via the ESX-1 type VII secretion system then expose bacterial DNA in the cytosol [23–25], leading to IFN-I secretion via cGAS–STING sensing and enhanced IL-1β secretion via NLRP3 inflammasome activation [25–27]. However, a recent study reported that IFN-I secretion varies depending on the *Mtb* strains despite the lack of differences in bacterial cytosol access via phagosomal rupture, suggesting that the presence of other factors affects IFN-I secretion in *Mtb* infection [27]. Indeed, the authors demonstrated that mitochondrial oxidative stress-mediated release of mtDNA into the cytosol plays a more crucial role in IFN-I secretion via the cGAS-STING axis than bacterial DNA in the cytosol [26, 27]. In NLRP3-dependent inflammasome activation, the contribution of cytosol-exposed oxidized mtDNA has also been proposed [28, 29].

Here, we sought to explore the role of mitochondrial oxidative stress as an upstream pathway in IFN-I production and NLRP3 inflammasome activation in MAB infections of murine macrophages. We found that increased mtROS induced by MAB-R infections lead to enhanced IFN-I and IL-1β production by the release of oxidized mtDNA into the cytosol and increased intracellular bacterial growth via cytosolic escape after phagosomal rupture.

## Results

### Live MAB-R strains enhanced mitochondrial oxidative stress compared to the effects of MAB-S strains

**Fig 1:**
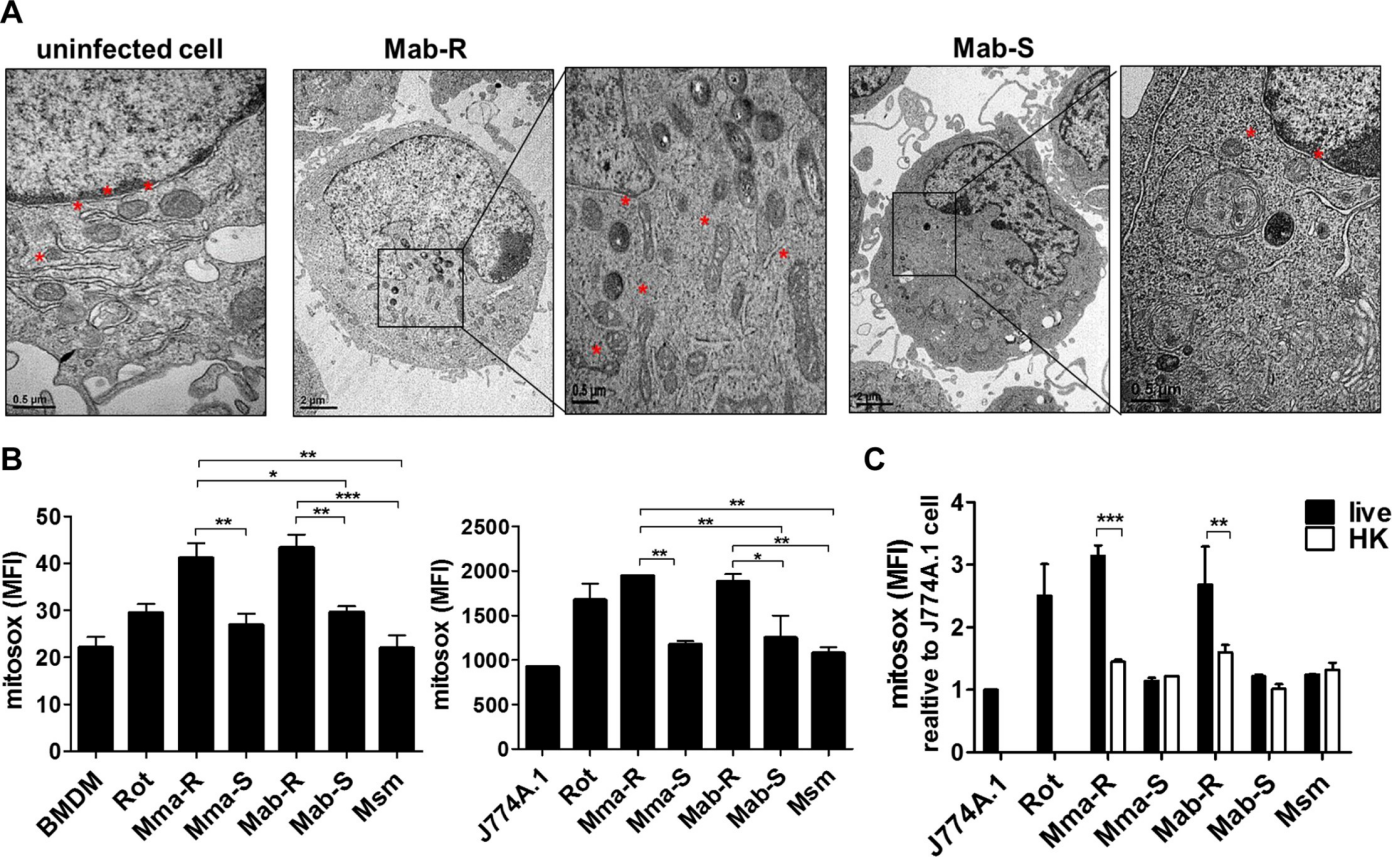
MAB-R strains enhanced mtROS in infected murine macrophages compared to the effects of MAB-S strains. (A) Representative TEM images of BMDMs infected with Mab-R or -S strains at an M.O.I. of 10 for 24 h. As a control, representative images of uninfected cells are shown. The mitochondrial morphology in infected cells and their magnified images are shown (black box region and marked with a red asterisk). Bar indicates 0.5 or 2 μm. (B) To evaluate mtROS (MitoSOX), BMDMs or J774A.1 cells were infected with strains of MAB-R, MAB-S or *M*. *smegmatis* (Msm) at an M.O.I. of 10 for 24 h. Additionally, cells were pre-treated with rotenone (Rot; 5 μM) as a positive control (for induction of ROS) for 30 min. The infected cells were stained with MitoSOX and then analysed by flow cytometry (FACSCalibur). (C) Induced mtROS levels in J774A.1 cells infected with live or heat-killed (HK) mycobacteria (M.O.I. of 10) for 24 h were measured by flow cytometry (FACSCalibur). Error bars represent the SD. Statistical significance was determined by ANOVA with Tukey's multiple comparison test (B and C) and two-tailed Student’s *t*-test (D).

Previously, we reported that active replication of MAB-R strains within phagosomes led to phagosomal rupture [22], which causes subsequent mitochondrial damage [30–32]. To examine whether infection with a rough type of *M*. *abscessus* strain (MAB-R) in murine BMDMs also induced mitochondrial damage, we observed mitochondrial morphology by transmission electron microscopy (TEM). TEM images showed that normal mitochondria in uninfected macrophages were geometrically spheroid-shaped or had reticulated morphologies. In contrast, mitochondria in macrophages infected with the MAB-R strain were shrunken or distorted and lengthened. However, mitochondrial morphology in macrophages infected with a smooth type of *M*. *abscessus* strain (MAB-S) was similar to that of normal mitochondria, and the mitochondrial damage was relatively minor compared to that in cells infected with MAB-R strains (Fig 1A). We next examined the involvement of mtROS. The levels of mtROS were increased in murine macrophages infected with two different MAB-R strains [Mma-R (*M*. *massiliense*, rough strain) and Mab-R (*M*. *abscessus* type strain ATCC 19977, rough strain)] (Fig 1B). Furthermore, to determine whether mtROS induction by MAB-R strains was dependent on active multiplication, we compared the mtROS levels induced by live or heat-killed (HK) MAB-R strains. Interestingly, live but not HK MAB-R strains significantly induced mtROS. However, MAB-S strains and *M*. *smegmatis* (Msm) did not induce mtROS, regardless of whether the mycobacteria were live or HK (Fig 1C). These results suggest that active multiplication of MAB-R strains contributes to mitochondrial oxidative stress in infected murine macrophages. This trend was also found in J774A.1 cells infected with MAB-R and -S strains of various clinical genotypes (S1 Fig).

### The mtROS induced by MAB-R strains increased the release of oxidized cytosolic mtDNA

**Fig 2:**
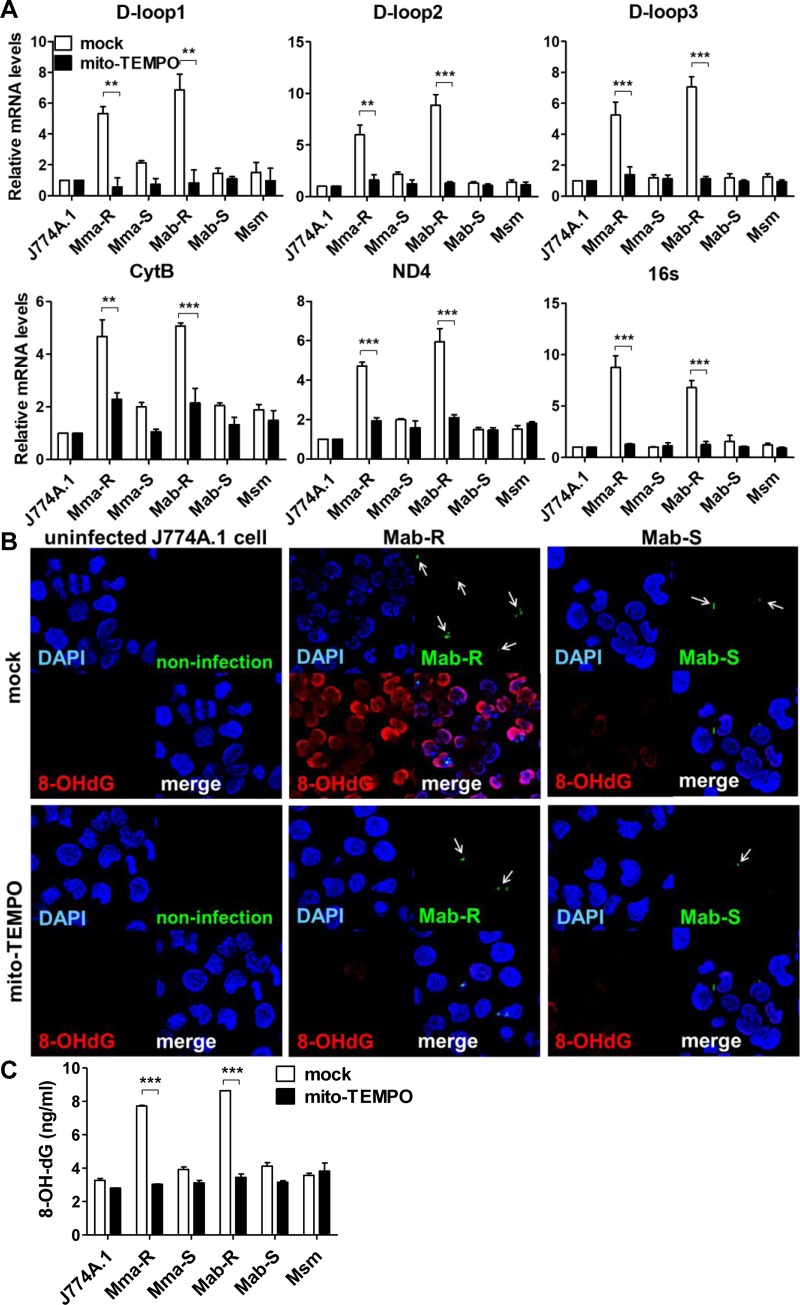
MAB-R strains led to increased cytosolic oxidized mtDNA in an mtROS-dependent manner in infected murine macrophages. (A) Cytosolic mtDNA was extracted from the nuclear and cytosolic fractions of J774A.1 cells that were infected with MAB-R, MAB-S, or *M*. *smegmatis* (Msm) (M.O.I. of 10) and pre-treated with or without mito-TEMPO (100 μM) for 24 h. Measurement of cytosolic mtDNA expression by qRT-PCR using the mitochondrial D-loop (D-loop-1, -2, and -3), CytB, ND4 and 16S primer sets. Normalization was performed as described in the materials and methods. (B) Representative confocal microscopic images of 8-OHdG induction in infected cells. J774A.1 cells were pre-treated with mito-TEMPO and infected with CFSE-labelled Mab-R or -S (green) at an M.O.I. of 10 for 24 h. Then, immunofluorescence staining using anti-8-oxyhydrodioxy guanosine (8-OHdG), a marker of DNA oxidative damage (red) was performed, and macrophage nuclei were stained with DAPI (blue). All images were captured at 100× magnification. (C) J774A.1 cells were infected with strains of MAB-R, MAB-S or *M*. *smegmatis* (Msm) at an M.O.I. of 10 for 24 h with or without mito-TEMPO treatment. Cytosolic DNA was obtained from infected cells, and the levels of 8-OHdG were measured by using an ELISA kit. Error bars represent the SD. Statistical significance was determined by two-tailed Student’s *t*-test (A and C).

mtROS can trigger the release of mtDNA into the cytosol and induce the NLRP3 inflammasome [28, 33]. We therefore measured cytosolic mtDNA levels in macrophages infected with MAB-R and -S strains via qRT-PCR. Our data showed that all six cytosolic mtDNA genes tested (D-loop-1, -2, and -3, CytB, ND4 and 16S) were highly expressed in macrophages infected with MAB-R strains compared to those of macrophages infected with MAB-S strains and even *M*. *marinum*, which is known to be capable of phagosomal escape (S2A Fig) [34, 35]. We also sought to determine whether mtROS affected the release of mtDNA into the cytosol in cells infected with MAB-R strains. We measured the levels of cytosolic mtDNA in the presence of mito-TEMPO (a mitochondrial-targeted antioxidant). We found that treatment with mito-TEMPO decreased the release of all cytosolic mtDNA genes tested (D-loop-1, -2, and -3, CytB, ND4 and 16S) in MAB-R-infected J774A.1 cells but not in cells infected with MAB-S strains or Msm (Fig 2A).

It has been reported that mtROS generation results in oxidized mtDNA, which increases mitochondrial 8-hydroxy-2-deoxyguanosine (8-OHdG), a marker of oxidative DNA damage in the cytosol of cells [28, 33]. To verify that increased cytosolic oxidized mtDNA was induced by mtROS in MAB-R-infected cells, we performed immunofluorescence staining for the accumulation of 8-OHdG. MAB-R-infected J774A.1 cells showed markedly increased 8-OHdG fluorescence intensity, but the 8-OHdG signal was very low in MAB-S-infected cells. Additionally, the 8-OHdG signal induced by MAB-R was markedly decreased by treatment with mito-TEMPO (Fig 2B). We next quantified 8-OHdG in cytosolic mtDNA from MAB-R- or MAB-S-infected cells via 8-OHdG ELISA. Our data also showed that the levels of 8-OHdG in the cytosol mtDNA of MAB-R-infected cells were also significantly decreased by treatment with mito-TEMPO (Fig 2C). Cyclosporine A (CsA, a mitochondrial pore inhibitor) treatment also resulted in a similar effect on 8-OHdG immunofluorescence staining and 8-OHdG ELISA (S2B and S2C Fig). Collectively, these results indicate that mtROS induced by MAB-R infections enhanced the release of oxidized mtDNA into the cytosol in infected murine macrophages.

### The mtROS induced by MAB-R strains increased IFN-I and IL-1β production in murine macrophages

**Fig 3:**
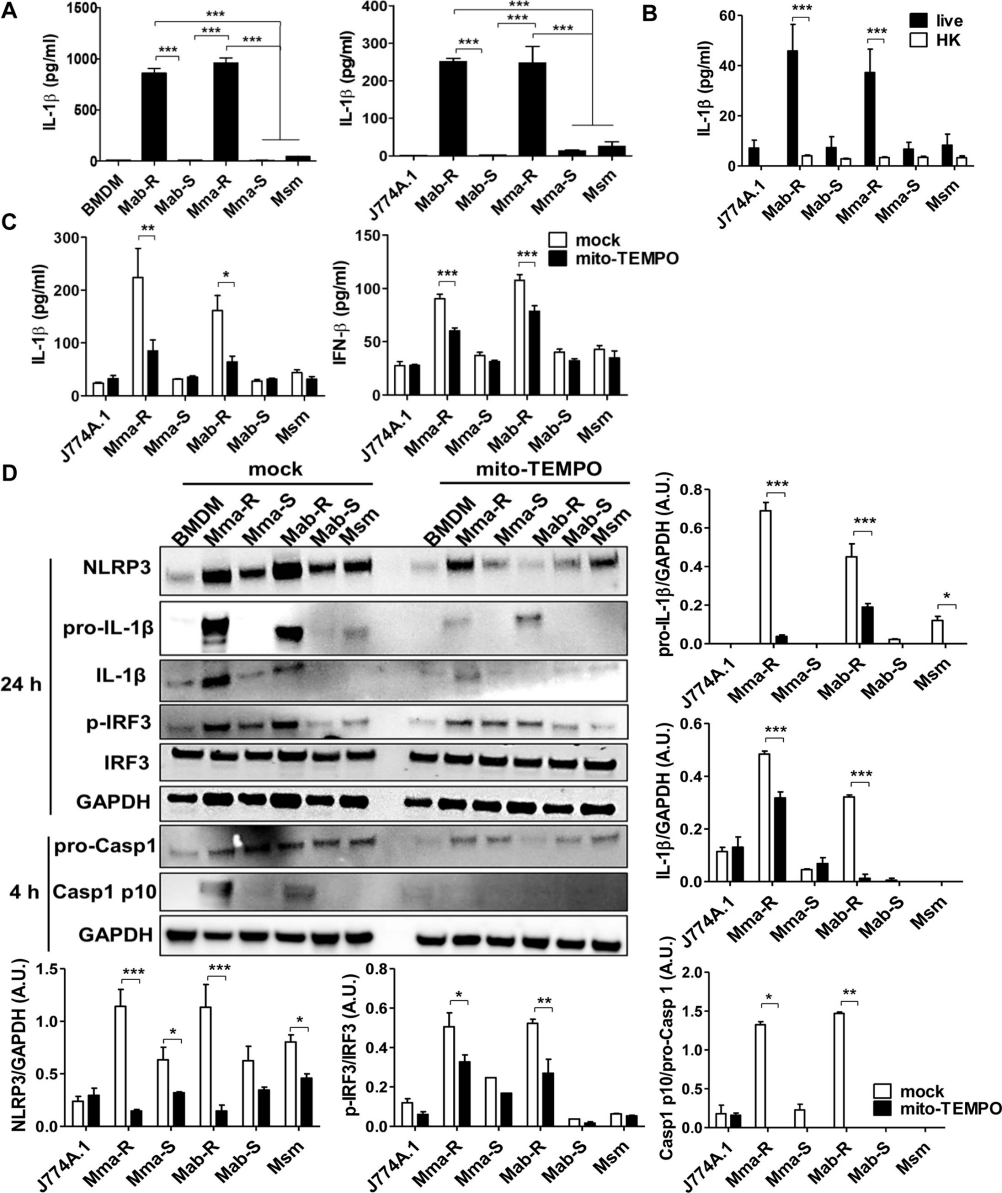
MAB-R-induced mtROS increased IFN-β and IL-1β production via activation of IRF-3 and the NLRP3 inflammasome. (A) BMDMs or J774A.1 cells were infected with strains of MAB-R, MAB-S or *M*. *smegmatis* (Msm) at an M.O.I. of 10 for 24 h. The supernatants of the infected cells were collected, and IL-1β levels were measured by ELISA. (B) Supernatants from J774A.1 cells infected with live or heat-killed (HK) strains of MAB-R, MAB-S or *M*. *smegmatis* (Msm) at an M.O.I. of 10 for 24 h were collected, and the levels of IL-1β were measured by ELISA. (C) J774A.1 cells were pre-treated with or without mito-TEMPO (100 μM) and infected with strains of MAB-R, MAB-S or *M*. *smegmatis* (Msm) at an M.O.I. of 10 for 24 h. IL-1β and IFN-β cytokine levels in the supernatants of infected cells were analysed by ELISA. (D) Representative immunoblot images and quantitative bar graphs of NLRP3 (110 kDa), IL-1β (pro form, 35 kDa; active form, 17 kDa), p-IRF3 (44~55 kDa), IRF3 (44~55 kDa) and caspase-1 (Casp1; pro form, 45 kDa; active form, 10 kDa) induced by the indicated mycobacteria (M.O.I. of 10) infection for 4 or 24 h in BMDMs pre-treated with or without mito-TEMPO. The expression levels of IL-1β, NLRP3 and caspase-1 were normalized to that of GAPDH. Error bars represent the SD. Statistical significance was determined by ANOVA with Tukey's multiple comparison test (A) and two-tailed Student’s *t*-test (C and D).

Other studies have indicated that mtROS play an important role in NLRP3 inflammasome activation [36, 37]. Next, we analysed whether MAB-R induced increased IL-1β secretion. We found that MAB-R strains increased IL-1β secretion in two types of macrophages (BMDM and J774A.1 cells) compared to those of macrophages infected with MAB-S strains or Msm (Fig 3A). A similar trend was also found in J774A.1 cells infected with MAB clinical strains of various genotypes (S3A Fig). We also investigated IL-1β production induced by live and HK mycobacteria. Of interest, live MAB-R but not HK strains enhanced IL-1β secretion in macrophages (Fig 3B), suggesting that IL-1β secretion induced by MAB-R strains was dependent on active replication.

Next, we investigated whether inhibition of mtROS by treatment with mito-TEMPO or CsA affected the expression of the cytokines IFN-I and IL-1β in MAB-R-infected cells. First, we determined whether the mtROS induced by MAB-R strains was decreased by mito-TEMPO or CsA (S3B Fig). Our data showed that treatment with mito-TEMPO or CsA inhibited IL-1β and IFN-β production in MAB-R-infected cells (Fig 3C and S3C Fig). A similar trend was also observed in IL-6 production in infected J774A.1 cells, suggesting that IFN-I, IL-1β and IL-6 production in cells infected by MAB-R strains depends on mtROS induction (S3D Fig). However, treatment with mito-TEMPO or CsA did not affect the production of TNF-α and IL-10, suggesting that their production does not depend on mtROS induction (S3D Fig).

Consistent with the above findings, immunoblots of cell lysates from infected BMDMs harvested at 24 h showed that MAB-R strains enhanced expression of pro-IL-1β and the NLRP3 inflammasome compared to that of BMDMs infected with MAB-S strains, suggesting that enhanced expression of pro-IL-1β and the NLRP3 inflammasome contributes to the increase in IL-1β secretion induced by MAB-R strains. Moreover, we also found that MAB-R strains enhanced production of mature IL-1β (17-kDa) protein as well as the expression of pro-IL-1β (35-kDa) in infected cells compared to those in cells infected with MAB-S strains or Msm (Fig 3D), suggesting that not only the expression of the NLRP3 inflammasome but also its activation contribute to the increase in IL-1β secretion induced by MAB-R strains. The elevated production of mature caspase-1 (10-kDa) found in cells infected with MAB-R strains further supported this finding (Fig 3D). We also found that NLRP3, IL-1β, p-IRF3 and caspase-1 p10 in MAB-R-infected BMDMs were significantly reduced by treatment with mito-TEMPO (Fig 3D), suggesting that enhanced IFN-I and IL-1β production induced by MAB-R is dependent on mtROS.

### mtROS enhanced intracellular survival of MAB-R strains via bacterial cytosol escape after phagosomal rupture

Previously, we reported that MAB-R strains led to cytosolic escape of bacteria following active replication within phagosomes, which contributes to bacterial intracellular survival via IFN-I-dependent cell-to-cell spreading [22]. We therefore examined whether mtROS, an upstream signal of IFN-I production, affected bacterial cytosolic escape via phagosomal rupture in MAB-R-infected macrophages. We randomly selected 15 phagosomes in MAB-R-infected J774A.1 cells and then compared the bacterial numbers in each phagosome. Confocal microscopy showed that treatment with mito-TEMPO significantly inhibited bacterial replication in MAB-R-infected macrophages after 24 h of infection (S4 Fig). However, there were no significant differences in bacterial number between mito-TEMPO treatment and no treatment 4 h after infection.

**Fig 4:**
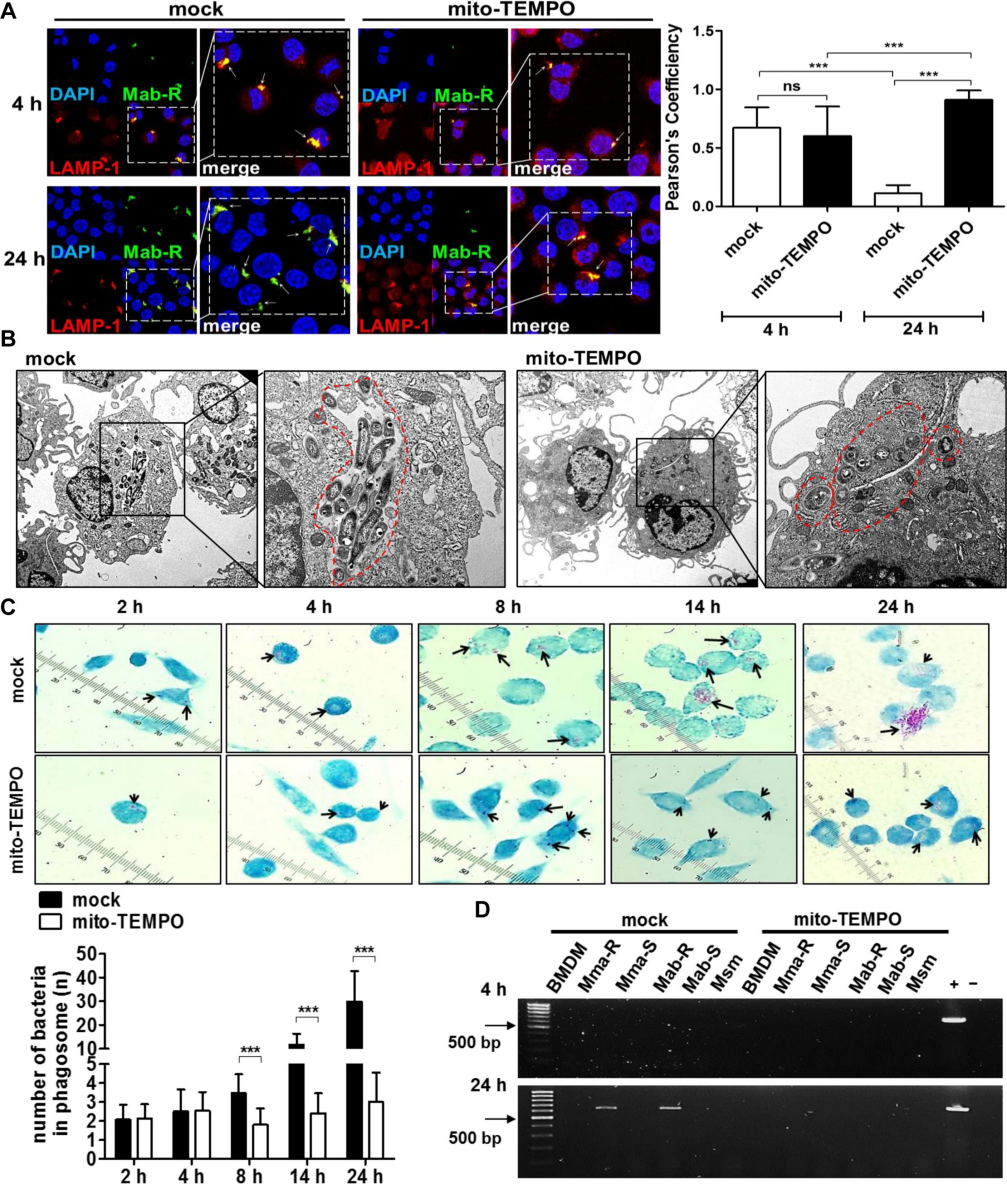
MAB-R infection-induced mtROS led to intracellular bacterial growth via enhanced cytosolic escape of the bacteria. (A) J774A.1 cells were pre-treated with mito-TEMPO (100 μM) and infected with CFSE-labelled (green) Mab-R at an M.O.I. of 10 for 4 or 24 h. Then, infected cells were stained with LAMP -1 (red) and DAPI (blue). Representative images are shown for LAMP-1 (red) and Mab-R (green) co-localization. Bar graph of Pearson's correlation coefficient values for the co-localization of MAB-R and LAMP-1. Twenty randomly selected bacteria were analysed and are representative of two independent experiments. (B) Representative TEM images of Mab-R (M.O.I. of 10 for 24 h) infected BMDMs with or without mito-TEMPO treatment are shown. Red dashed lines indicate the Mab-R-containing phagosomes. (C) Untreated or mito-TEMPO-pre-treated J774A.1 cells infected with Mab-R at an M.O.I. of 10 for different times (2, 4, 8, 14 and 24 h). The infected cells were analysed by AFB staining and observed under a microscope at 100× magnification. The black arrows indicate MAB-R (red-stained bacilli) in methylene blue-stained J774A.1 cells. The bar graph shows the result of intracellular bacterial numbers in each phagosome by randomly counting 15 phagosomes in MAB-R-infected cells. (D) Mito-TEMPO-treated or untreated J774A.1 cells infected with strains of MAB-R, MAB-S or *M*. *smegmatis* (Msm) at an M.O.I. of 10 for 4 or 24 h. Cytosolic DNA was extracted using cellular fractionation and the phenol-chloroform-isoamyl alcohol (PCI) method from J774A.1 cells infected with the indicated bacteria. Cytosolic mycobacterial DNA was detected by PCR amplification of the hsp65 (603 bp) gene (non, uninfected cells; +, mycobacterial DNA; −, contains only primers). Error bars represent the SD. Statistical significance was determined by two-tailed Student’s *t*-test (A and C).

Our previous study also showed that MAB-R strains but not MAB-S showed reduced co-localization signals with LAMP-1, a late endosome marker [22, 38], due to their cytosolic escape after phagosomal rupture. In this study, we also analysed the co-localization of MAB-R strains with LAMP-1 by confocal microscopy in infected macrophages in the presence of mito-TEMPO. We found that treatment with mito-TEMPO led to enhanced co-localization with LAMP-1 at 24 h but not 4 h after infection (Fig 4A). However, this was not true in cells that were not treated with mito-TEMPO. These results suggest that mtROS induced by MAB-R infection led to their intracellular survival via phagosomal rupture in murine macrophages. TEM (Fig 4B) and AFB staining analysis (Fig 4C and S5 Fig) also showed a positive role of mtROS in active bacterial replication within phagosomes in MAB-R-infected macrophages, further supporting our above findings.

To further determine whether mtROS leads to the escape of MAB-R strains into the cytosol during MAB-R infection, we conducted PCR analysis of the cytosolic fraction of MAB-R-infected macrophages. These results also indicated that mito-TEMPO treatment inhibited MAB-R escape into the cytosol in infected macrophages (Fig 4D and S6 Fig). Together, these results suggest that mtROS leads to active replication within the phagosome and bacterial cytosolic escape via phagosomal rupture in MAB-R-infected macrophages.

### mtROS enhanced intracellular survival of MAB-R strains via IFN-I-dependent cell-to-cell spreading and cell cytotoxicity

**Fig 5:**
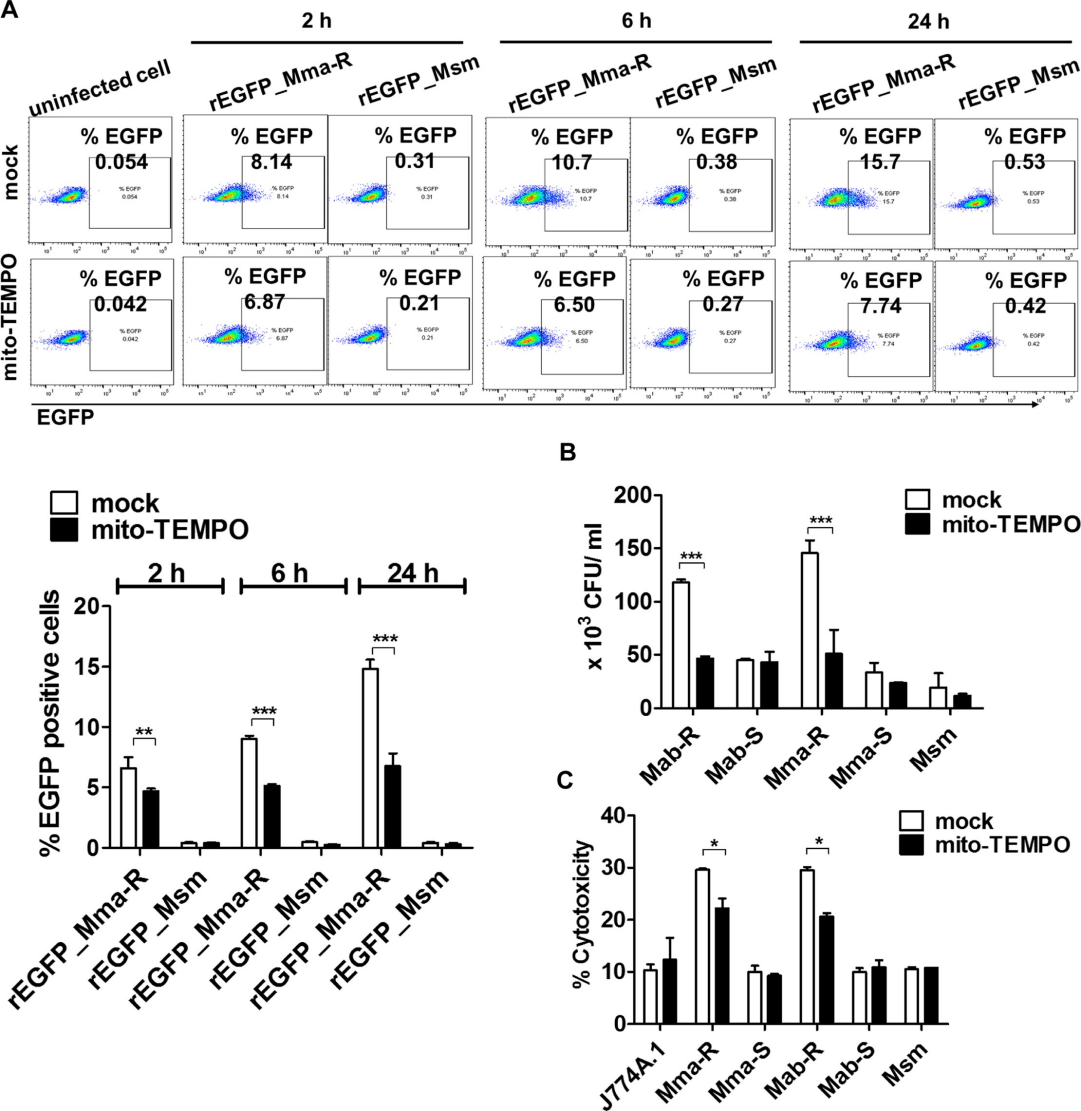
mtROS led to enhanced intracellular survival of MAB-R strains via IFN-I-dependent cell-to-cell spreading and enhanced cell cytotoxicity. (A) J774A.1 cells pre-treated with mito-TEMPO (100 μM) and infected with rEGFP_Mma-R or rEGFP_Msm (*M*. *smegmatis*) at an M.O.I. of 10 for different times (2, 6 and 24 h). The percentage of EGFP-positive cells was measured by flow cytometry (FACSCalibur). (B) J774A.1 cells were pre-treated with mito-TEMPO and infected with strains of MAB-R, MAB-S or *M*. *smegmatis* (Msm) at an M.O.I. of 10 for 24 h. Infected cell lysates were serially diluted and plated onto 7H10 agar plates for CFU assays. (C) Cytotoxicity was quantitated by measuring lactate dehydrogenase (LDH) in the infected cell supernatants. Error bars represent the SD. Statistical significance was determined by two-tailed Student’s *t*-test (A-C).

Next, to determine the effect of mtROS on IFN-I-dependent cell-to-cell spreading induced by infection with MAB-R strains, we analysed J774A.1 cells infected with recombinant rEGFP_Mma-R and rEGFP_Msm. Our previous study showed that MAB-R strains induced cell-to-cell spreading and cell cytotoxicity in infected macrophages in an IFN-I-dependent manner [22]. In this study, cell-to-cell spreading was inhibited by mito-TEMPO treatment (Fig 5A), suggesting that mtROS inhibits IFN-I-dependent cell-to-cell spreading by suppressing IFN-I production.

The role of mtROS in inhibiting cytosolic escape via phagosomal rupture and IFN-I-dependent cell-to-cell spreading leads to enhanced intracellular survival in MAB-R-infected macrophages. To address this issue, we first evaluated intracellular survival of MAB-R in the presence of mito-TEMPO or CsA via a CFU assay. We found that treatment with mito-TEMPO reduced the intracellular growth of MAB-R but not MAB-S, suggesting a positive role of mtROS in MAB-R intracellular survival (Fig 5B and S7A Fig). A similar trend was also found in cells infected with clinical isolates of various genotypes (S7C Fig). Second, to further determine the role of mtROS in intracellular survival at different times (2, 6 and 24 h), luciferase activities were compared in cells infected with two luciferase-expressing recombinant MAB-R (rLuci_Mma-R) and *M*. *smegmatis* (rLuci Msm) strains in the presence of mito-TEMPO (S7D Fig). Our data showed that the luciferase activity of MAB-R but not Msm was markedly inhibited after 24 h of infection in mito-TEMPO-treated J774A.1 cells. However, there were no significant differences in luciferase activity between mito-TEMPO treatment and no treatment at 2 h or 6 h after infection. This suggests that the enhancing effect of mtROS on intracellular survival MAB-R may occur after bacterial escape into the cytosol following phagosomal rupture.

Our previous study also showed that IFN-I induced by MAB-R infection enhanced the cytotoxicity of infected macrophages [22]. Therefore, to determine the role of mtROS in MAB-R-mediated cytotoxicity, we analysed the effect of mtROS on the cytotoxicity of macrophages infected with MAB-R, MAB-S or Msm via lactate dehydrogenase (LDH) assays. In cells infected with MAB-R but not MAB-S or Msm strains, the cytotoxicity of infected cells was decreased in the presence of mito-TEMPO or CsA (Fig 5C and S7B Fig), suggesting a positive role of mtROS in the cytotoxicity of MAB-R-infected cells. Collectively, our data suggest that mtROS induced by MAB-R infection plays a pivotal role in intracellular bacterial growth via bacterial escape into the cytosol and IFN-I-mediated cell-to-cell spreading in murine macrophages. Furthermore, MAB-R infection contributes to cell stress-mediated cytotoxicity.

### MAB-R infections enhanced IFN-I production via the cGAS-STING pathway

**Fig 6:**
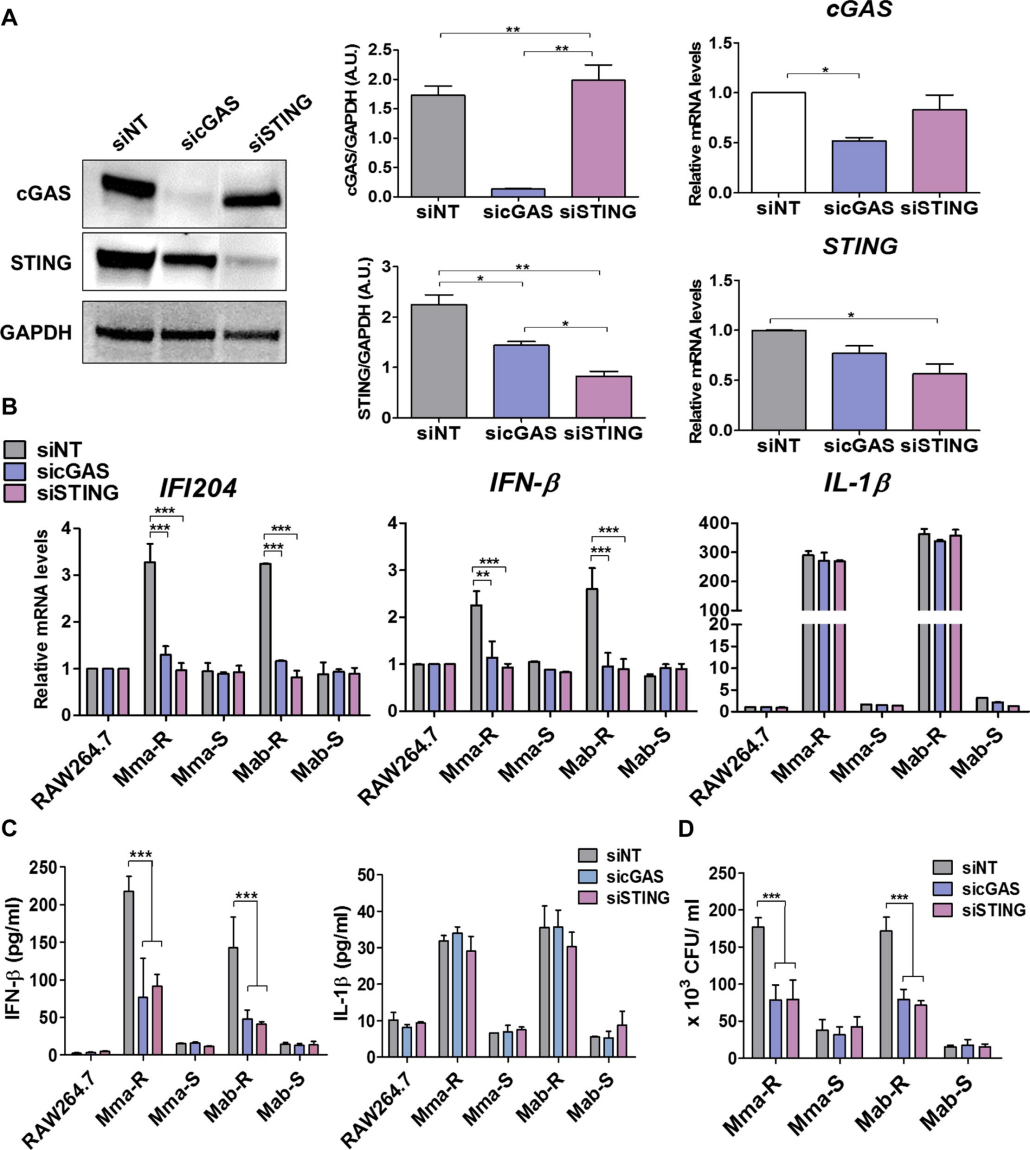
MAB-R infection enhanced IFN-β production via the cGAS−STING axis in infected murine macrophages. (A) RAW264.7 cells were transfected with siRNAs targeting cGAS or STING or a scramble siRNA (siNT). Forty-eight hours after transfection, protein and transcription levels were measured in total cell lysates by immunoblotting (left panels) and RT-qPCR (right panels), respectively. (B-D) RAW264.7 cells were transfected with cGAS, STING or a scramble siRNA (siNT). Forty-eight hours after transfection, the cells were infected with MAB-R or -S strains (10 M.O.I) and then harvested 24 h after infection. The transcription levels of IFN-β and IL-1β in infected cells were measured via qRT-PCR. Normalization was performed using the β-actin gene (B). IL-1β and IFN-β cytokine levels in the supernatant of infected cells were measured by ELISA (C), and infected cell lysates were serially diluted and plated onto 7H10 agar plates for CFU assays (D). Error bars represent the SD. Statistical significance was determined by ANOVA with Tukey's multiple comparison test (A) two-way ANOVA with multiple comparison test (B-D).

It has been reported that cytosolic oxidized mtDNA leads to IFN-I production via the cGAS-STING pathway [26, 39]. Here, we examined whether IFN-I production by MAB-R infection was dependent on the cGAS-STING pathway. First, we verified that siRNA knockdown of cGAS or STING resulted in reductions in expression of each gene in siRNA-transfected RAW264.7 cells. (Fig 6A). Our data showed that the levels of transcription and secretion of IFN-β in MAB-R-infected cells were significantly decreased following cGAS or STING siRNA treatment compared to those of scramble treatment (Fig 6B and 6C), suggesting that enhanced IFN-I signalling in MAB-R-infected cells depends on the cGAS-STING pathway. However, cGAS or STING siRNA treatment did not affect the levels of transcription and secretion of IL-1β in MAB-R-infected cells (Fig 6B and 6C), suggesting that the enhanced IL-1β production found in MAB-R-infected cells was independent of the cGAS-STING pathway. Furthermore, we also found that MAB-R strains subjected to cGAS or STING knockdown had significantly decreased intracellular bacterial growth (Fig 6D). Together, these results suggest that MAB-R infection enhances IFN-I production in murine macrophages in a cGAS-STING-dependent manner, but NLRP3 inflammasome-mediated IL-1β production is not affected.

### Crosstalk between IFN-I and IL-1β in MAB-R-infected murine macrophages

**Fig 7:**
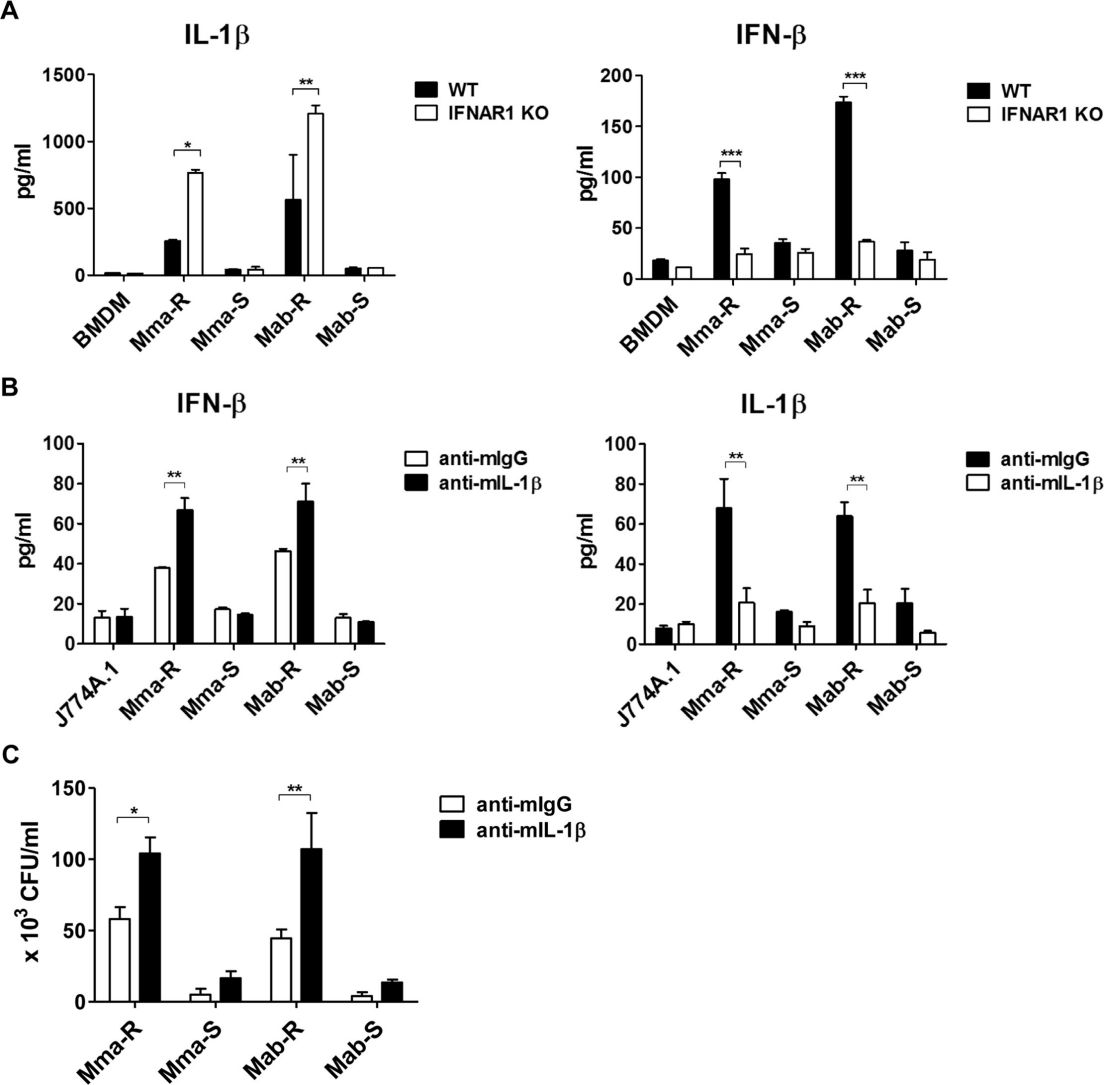
Crosstalk between IFN-I and IL-1β in MAB-R infection of murine macrophages. (A) BMDMs were obtained from wild-type (WT) or IFNAR1 KO mice and infected with MAB-R and -S strains at an M.O.I. of 10 for 24 h. Supernatants from the infected cells were collected, and IL-1β and IFN-β cytokine levels were measured by ELISA. (B-C) BMDMs were treated with anti-mouse IgG (10 μg/ml) or anti-mouse IL-1β receptor (10 μg/ml) neutralizing antibodies. Then, the cells were infected with MAB-R and -S stains at an M.O.I. of 10 for 24 h. IL-1β and IFN-β cytokine levels in the infected cell supernatants were measured by ELISA (B), and infected cell lysates were serially diluted and plated onto 7H10 agar plates for CFU assays (C). Error bars represent the SD. Statistical significance was determined by two-tailed Student’s *t*-test (A-C).

Next, to evaluate the relationships between IL-1β and IFN-I production in MAB-R infection, the levels of IL-1β and IFN-β were examined in BMDMs from interferon receptor-1 knockout (IFNAR1 KO) mice or in J774A.1 cells subjected to interleukin-1 receptor (IL-1R1) blocking antibodies (Abs). Our results showed that the level of IL-1β production was significantly increased in MAB-R-infected BMDMs from IFNAR1 KO mice compared to those of wild-type mice (Fig 7A). Similarly, IFN-β secretion induced by MAB-R strains was markedly increased in J774A.1 cells in which the IL-1β receptor was blocked (Fig 7B). Moreover, inhibition of the IL-1β receptor increased intracellular bacterial growth of MAB-R strains (Fig 7C). Collectively, our results suggest that reciprocal inhibition exists between IL-1β and IFN-I production and that IL-1β exerts an anti-bacterial effect in MAB-R infection of murine macrophages, consistent with previous reports [22, 25, 40, 41].

## Discussion

Recently, we reported that MAB-R, but not MAB-S strains, escape into the cytosol via phagosomal rupture in infected murine macrophages [22]. The bacteria escaped into the cytosol, led to enhanced IFN-I production and cell death-mediated cell-to-cell spreading, resulting in enhanced bacterial survival. It highlights a pivotal role of phagosomal rupture-mediated IFN-I, providing a likely explanation into the higher virulence of MAB-R strains compared to that of MAB-S strains [22]. As an extension of our previous study, we examined the role of mtROS in IFN-I secretion, inflammasome activation, and intracellular bacterial survival in MAB-R-infected murine macrophages. There are several noteworthy findings regarding the role of mtROS in MAB pathogenesis.

It has been shown in *Mtb* infections that intracellular pathogens lead to mitochondrial damage in infected macrophages, which induces the subsequent release of mtROS and mtDNA into the cytosol [27]. Similarly, in this study, for the first time, we demonstrated that MAB-R but not MAB-S strains enhanced mtROS production (Fig 1B and S1 Fig). These effects led to mtROS-mediated release of oxidized mtDNA into the cytosol of infected murine macrophages, which was proven by our data showing increases in six cytosolic mtDNA genes (D-loop-1, -2, and -3, CytB, ND4, and 16S) and increased 8-OHdG in macrophages infected with MAB-R versus other mycobacteria (Fig 2B and 2C and S2 Fig).

Cytosolic oxidized mtDNA enhances IFN-I production by activating a DNA-dependent cytosolic surveillance pathway (CSP) called the cGAS-STING axis [28, 42–45]. We found that cytosolic oxidized mtDNA enhanced IFN-I production in a cGAS-STING pathway-dependent manner in MAB-R-infected murine macrophages (Figs 3 and 6). Consistent with these results, we also demonstrated that treatment with mito-TEMPO or CsA, which are capable of modulating mtROS, inhibited the induced IFN-I production in murine macrophages (Fig 3 and S3C Fig). Moreover, mito-TEMPO also inhibited IFN-I-mediated cell-to-cell spreading and cytotoxicity, resulting in reduced intracellular survival of MAB-R strains (Fig 5). Our data suggest that mtROS positively regulate IFN-I production as an upstream signal in MAB-R-infected murine macrophages.

In addition to IFN-I production, cytosolic oxidized mtDNA also enhances IL-1β production via directly activating the NLRP3 inflammasome [27, 28]. These effects contribute to the pathogenesis of intracellular bacteria such as *Mtb* by dampening inflammation [46, 47]. In this study, we found that oxidized mtDNA led to NLRP3 inflammasome-mediated IL-1β production in MAB-R-infected murine macrophages, which was mediated by the enhanced expression of pro-IL-1β and NLRP3 and by inflammasome activation (Fig 3). Consistently, mito-TEMPO or CsA negatively regulated IL-1β production in murine macrophages by inhibiting the NLRP3 inflammasome (Fig 3C and 3D and S3C Fig), indicating that mtROS positively regulate not only INF-I but also IL-1β as upstream signals in MAB-R-infected murine macrophages. This suggests that mtROS play a pivotal role in the inflammation-mediated pathogenesis of MAB-R as a kind of specific class of PAMP.

We recently reported that IFN-I facilitates intracellular bacterial replication in MAB-R-infected murine macrophages [22]. Therefore, we also examined the role of the inflammasome in intracellular bacterial survival. Our data showed that blocking the IL-1β receptor (IL-1R1) in J774A.1 cells enhanced bacterial survival compared to that of J774A.1 cells treated with normal IgG (Fig 7C). This suggests that in contrast to IFN-I, inflammasomes exert an antibacterial effect in MAB-R-infected murine macrophages. We also evaluated the crosstalk between IFN-I and the inflammasome during MAB-R infection in murine macrophages. Several recent studies have shown that IFN-I inducible proteins also act as negative regulators inhibiting inflammasome activation [47–49]. IFN-I signalling inhibits activity of the NLRP3 inflammasome via the STAT-1 axis [48, 49]. In addition, it has also been reported that IFN-I mediated inducible nitric oxide synthase (iNOS) and NO production inhibit NLRP3 oligomerization by means of direct S-nitrosylation of the NLRP3 protein, preventing full inflammasome assembly [41]. Consistent with the previous findings, we also found that MAB-R infection increased IL-1β production in BMDMs from IFNAR1 KO mice compared to that of BMDMs from wild-type mice (Fig 7A), suggesting that IFN-I also contributes to intracellular MAB-R survival in infected macrophages by inhibiting inflammasome activation capable of exerting an antibacterial effect. Recently, we reported that MAB-R strains also induce phagosomal rupture in infected murine macrophages via disruption of the phagosome membrane by active replication that surpassed the phagosome accommodation capacity [22], which is distinct from the ESX-1-mediated phagosomal rupture of *Mtb* [23]. Therefore, in this study, we examined the role of mtROS in phagosomal rupture, bacterial cytosolic escape and intracellular replication in MAB-R infection. Unexpectedly, we found that mtROS induced by infection with MAB-R strains acted as an upstream signal for phagosomal rupture and bacterial cytosolic escape (Fig 4A, 4B and 4D) and facilitated intracellular bacterial replication in murine macrophages (Fig 4C, S4 and S5 Figs). Several recent studies reported that in infections with some intracellular bacteria, IFN-I response genes can work synergistically to rupture vacuoles containing bacteria that have entered the cytoplasm, resulting in the exposure of bacteria DNA to CSPs [50, 51]. Our present findings showed that mtROS induced IFN-I production as an upstream signal and facilitated phagosomal rupture-mediated cytosolic escape of bacteria in MAB-R-infected cells (Fig 4), suggesting that mtROS exert a pro-bacterial effect via induction of IFN-I in MAB-R-infected macrophages. Therefore, it is tempting to hypothesize that mtROS could compromise the integrity of the phagosomal membrane in an IFN-I-dependent manner, which could lead to intracellular bacterial replication at a level beyond that of the membrane capacity of the phagosome, allowing bacterial escape into the cytosol. The exact mechanism regarding the pro-bacterial effect of mtROS should be elucidated in the future.

**Fig 8:**
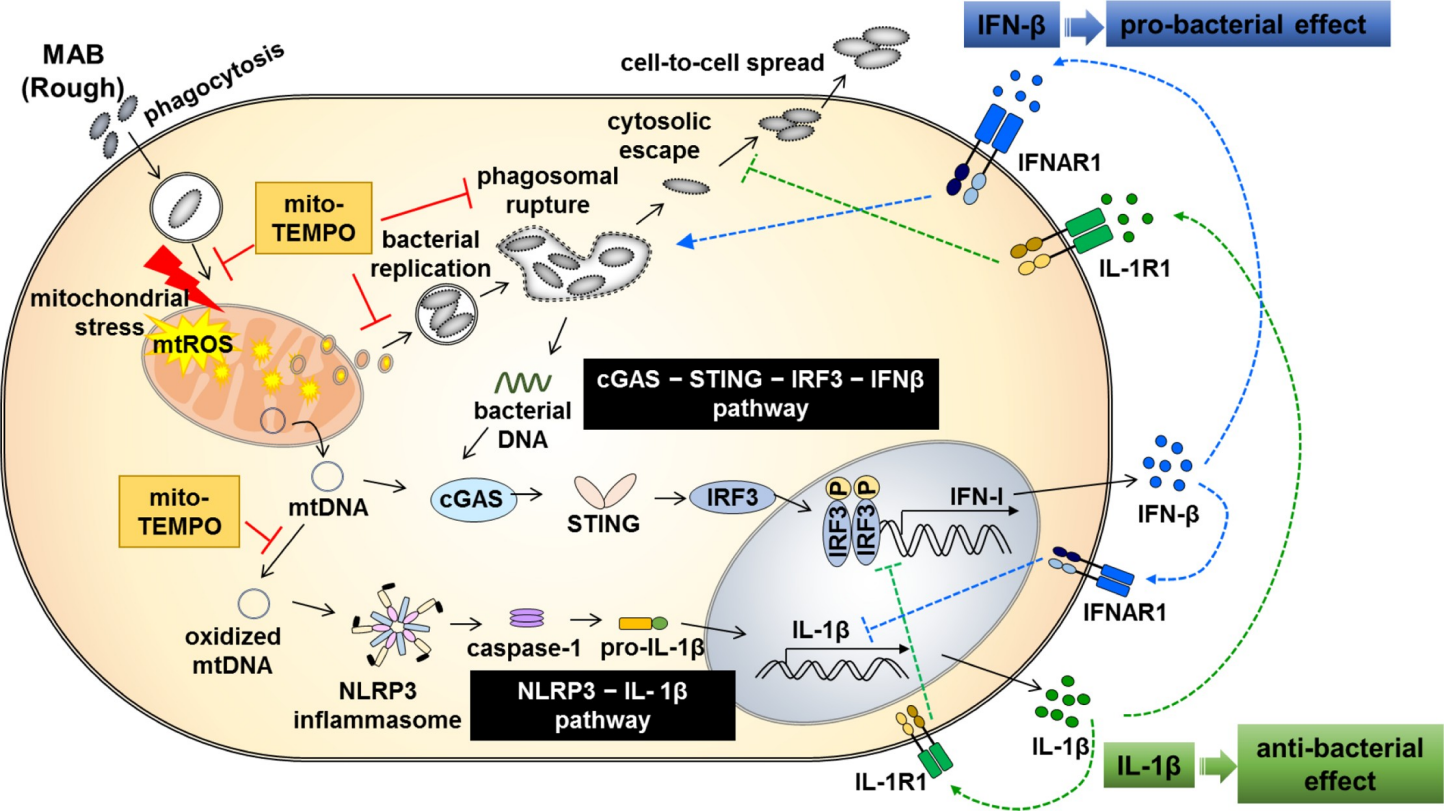
Schematic diagram showing mitochondrial oxidative stress induced by MAB-R infection of murine macrophages. MAB-R enters macrophages by phagocytosis and leads to mitochondrial oxidative stress. Our results suggest that MAB-R infection elicits both the i) cGAS−STING−IRF3−IFN-β and ii) NLRP3 inflammasome-IL-1β pathways in murine macrophages: i) Excess formation of mtROS leads to phagosomal rupture-mediated bacterial escape into the cytosol. Cytosolic mitochondrial and mycobacterial DNA induce IFN-I production via the cGAS−STING−IRF3 pathway. ii) Additionally, oxidized mtDNA released into the cytosol leads to activation of the NLRP3 inflammasome. However, inhibition of mtROS by treatment with mito-TEMPO or CsA decreases the levels of mtROS and also leads to decreased IFN-I and IL-1β production. In addition, inhibition of mtROS leads to a reduction in MAB-R replication in phagosomes and inhibition of phagosomal rupture, which ultimately reduces bacterial survival in infected murine macrophages. These two pathways induce opposite results for host defence against MAB-R infection. IL-1β is considered to have antibacterial effects, while IFN-I is recognized to be highly detrimental to bacterial replication.

In summary, in combination with our previous report [22], our current data revealed relationships between mtROS, IFN-I secretion, inflammasome activation, and intracellular bacterial survival in MAB-R-infected murine macrophages in a sequential or an interconnected manner (Fig 8). First, live MAB-R infection leads to mtROS via an as-yet unknown mechanism in murine macrophages, resulting in the release of oxidized mtDNA into the cytosol. Second, cytosolic mtDNA leads to dampening inflammatory responses via NLRP3 inflammasome-mediated IL-1β and cGAS-STING-dependent IFN-I production in infected macrophages. Third, the mtROS/cytosolic mtDNA axis induces intracellular bacterial replication and bacterial escape into the cytosol after phagosomal rupture, potentially via compromising membrane integrity in an IFN-I-dependent manner. Finally, IFN-I negatively regulates activation of the NLRP3 inflammasome, preventing IL-1β production, which further contributes to enhanced bacterial survival.

In conclusion, in this study, we have shown that MAB-R but not MAB-S strains enhance IFN-I and IL-1β production via mtROS induction as a kind of specific class of PAMP, which contributes to MAB-R pathogenesis via enhanced cell survival in macrophages and dampens inflammation. In addition, we also suggest that treatment targeting stages involved in mtROS generation may be very effective for the control of MAB infections.

## Materials and methods

### Ethics statement

All animal experiments were performed in compliance with the institutional recommendations in the National Guidelines for the Care and Use of Laboratory Animals. All protocols were approved by the Institutional Animal Care and Use Committee (IACUC; Approval No. SNU-181025-1) of the Institute of Laboratory Animal Resources at Seoul National University.

### Mice

Male C57BL/6 mice (~25 g, 6 weeks old) were purchased from Orient-Bio (Seongnam, South Korea). IFNAR1 KO mice on a C57BL/6 background [22] were bred and housed in specific pathogen-free conditions at Seoul National University College of Medicine. All mice were 8 ~ 12 weeks of age at the beginning of the experiments.

### Cell culture

The murine macrophage cell lines J774A.1 and RAW264.7 and bone marrow-derived macrophages (BMDMs) were used in this study. J774A.1 and RAW264.7 cells were cultured in Dulbecco’s modified Eagle’s medium (DMEM) supplemented with 10% foetal bovine serum and penicillin/streptomycin (100 U/ml) and were maintained at 37°C in a humidified atmosphere with 5% CO_2_. Before infection, the cells were plated in 12-well plates at 2×10^5^ cells per well overnight. BMDMs were prepared as previously described [22]. In brief, BM cells from the femurs and tibia of mice were cultured in 12-well tissue culture plates (1×10^6^ cells per well) with complete BMDM medium (DMEM containing 10% FBS, 1% sodium pyruvate, 4 mM glutamine, and 1% penicillin-streptomycin (100 units/ml) supplemented with 10% L929 cell-conditioned media) and were maintained at 37°C in a humidified atmosphere with 5% CO_2_. On days 1 and 3, the medium was replaced, and on day 5, BMDMs were used in further experiments.

### Bacterial condition and growth

All mycobacterial strains were grown as previously described [22]. Bacteria were grown in Middle brook 7H9 (Difco) supplemented with 10% ADC (BD), 0.2% glycerol and 0.05% Tween-80, passed through a 27-gauge needle three to five times to remove bacterial clumps. Bacterial concentration was determined by measuring the optical density at 600 nm (OD 600) as a function of CFU/ml. To prepare various heat-killed mycobacterial strains, we inactivated the bacteria at 100°C in a boiling-water bath for 30 min. The mycobacterial strains representatively used in this study are as follows: 4 type strains of *Mycobacterium abscessus* [(ATCC 19977 ^T^ rough (Mab-R) and smooth type (Mab-S)], *Mycobacterium smegmatis* ATCC 19420^T^ and *Mycobacterium marinum* JCM 17638^T^ and 2 clinical isolates of *Mycobacterium abscessus* subsp. *massiliense* type I smooth (Mma-S), type II-rough (Mma-R). The other various genotype mycobacteria, *M*. *abscessus* rough strains (ABS-R); A10 and A26; *M*. *abscessus* smooth strains (ABS-S): A12 and A21; *M*. *massiliense* type I-rough strains (MAS_I-R): A16, A22 and A34; *M*. *massiliense* type II-rough strains (MAS_II-R): A4 and A50594; *M*. *massiliense* smooth strains (MAS-S): A15 and A51843, were used.

### Host cell infection procedure and CFU assay

All bacterial strains were grown until the stationary growth phase, washed with PBS, centrifuged and resuspended in DMEM. J774A.1 cells or BMDMs were infected with the indicated mycobacteria at the M.O.I. (multiplicity of infection) reported in each figure legend, and infected cells were washed 2 h post infection with PBS. To eliminate extracellular mycobacteria, cells were incubated in fresh culture medium with 50 μg/ml amikacin [22, 52, 53]. Infected cell culture supernatants were collected and stored in a deep freezer (at -70°C) to determine the cytokine level. For the CFU assay, infected cells were washed with PBS and detached with 0.5% Triton X-100 or by scraping after infection. The cell pellets were diluted in PBS and plated onto 7H10 agar plates (supplemented with OADC) to determine the CFU counts.

### Transmission electron microscopy

For TEM analysis of *M*. *abscessus*, infected cells were fixed as previously described [22]. Briefly, infected cells were fixed with a solution containing 2% paraformaldehyde (v/v) and 2.5% glutaraldehyde (v/v) in 0.05 M cacodylate-HCl buffer at pH 7.2 for 2~4 h. After fixation, the samples were processed following conventional procedures. The sections were observed using a JEOL transmission electron microscope (Japan) at an accelerating voltage of 80 kV.

### Confocal microscopy

Bacteria were stained using CFSE (Invitrogen) according to the manufacturer's protocol. J774A.1 cells were seeded on 2-chamber plates and infected with the stained bacteria at an M.O.I. of 10 for 4 or 24 h. Infected cells were washed with PBS, fixed with 4% paraformaldehyde for 10 min at room temperature, permeabilized with 0.1% Triton X-100 for 10 min and blocked with 1% BSA for 30 min at room temperature. The cells were labelled with rat anti-LAMP1 antibody (#25245; Abcam) or anti-8-oxyhydrodioxy guanosine (8-OHdG-Alexa Fluor 647, sc-393871; Santa Cruz Biotechnology), which is a marker of DNA oxidative damage, incubated for 24 h at 4°C and then labelled with goat anti-rat IgG (H+L) Alexa Fluor 633 conjugate at a dilution of 1: 1,000 for 45 min at room temperature. Nuclei were stained with 300 nM DAPI (#D1306, Invitrogen) for 5 min at room temperature. The cover slips were washed thoroughly with PBS and mounted on glass slides with Vectashield solution (Vector Laboratories) and visualized by fluorescence microscopy using a confocal laser scanning microscope system (Olympus-FV3000).

### Acid-fast bacillus staining

A total of 1×10^5^ J774A.1 cells were cultured on 2-chamber plates and infected with *M*. *abscessus* at an M.O.I. of 10 for different times (2, 4, 8, 14 and 24 h). Infected cells were processed as previously described [22], and the Ziehl-Neelsen staining method [54, 55] was used for microscopic detection. In brief, all samples were air dried and heat fixed. The samples were smeared with carbol fuchsin stain and continuously steamed for 5 min with low or intermittent heat; the samples were then rinsed with distilled water and smeared with 3% acid alcohol (v/v) for decolorization. The slides containing the samples were rinsed with distilled water again, and methylene blue was used to stain other cells for 30~45 seconds. The samples were rinsed thoroughly with distilled water and air-dried and examined under a microscope with a ×100 oil immersion objective.

### Immunoblotting assay

A total of 1 × 10^6^ BMDMs were infected with various bacteria at an M.O.I. of 10 for 24 h. Cells were lysed in RIPA buffer supplemented with complete EDTA-free protease inhibitor (Roche) and a phosphatase inhibitor cocktail (Roche), and infected lysates were collected for protein analysis. Protein concentration was measured by Bradford assay (Bio‐Rad). Protein lysates were separated by SDS-polyacrylamide gel electrophoresis and transferred onto nitrocellulose membranes. The membranes were blocked with blocking buffer containing 5% non-fat milk/PBS-Tween or 5% BSA/PBS-Tween at room temperature for 1 h and incubated with primary antibodies overnight at 4°C. For this study, the primary antibodies were anti-rabbit IRF3 (#4302; Cell Signaling), anti-rabbit p-IRF3 (#29047; Cell Signaling), anti-NLRP3 (#15101; Cell Signaling), anti-rabbit IL-1β (#9722; Abcam), anti-mouse caspase-1 (sc-50636; Santa Cruz Biotechnology), anti-mouse caspase-1 p10 (sc-514; Santa Cruz Biotechnology), cGAS (#31659; Cell Signaling), STING (#13647; Cell Signaling) and anti-rabbit GAPDH (sc-25778; Santa Cruz Biotechnology). After three washes were performed with washing buffer, the membranes were incubated with secondary HRP-conjugated antibodies at room temperature for 1 h. After washing, the protein bands were visualized with chemiluminescent reagents following the manufacturer’s instructions (Amersham Imager 600 imagers, GE Healthcare) and quantified with Image J software.

### Interleukin-1 beta receptor blocking by antibody neutralization assay

For interleukin-1 beta receptor (IL-1R1) blocking, J774A.1 cells or BMDMs were seeded in 24-well plates at 2×10^5^ cells per well and incubated in complete culture media containing a constant amount of 10 μg/ml normal goat IgG control (#AB-108-C, R&D System Inc.) and mouse IL-1β (#AF-401-NA, R&D Systems Inc.). Then, the cells were infected with the indicated mycobacteria at an M.O.I. of 10 for 24 h. IL-1β secretion in the supernatant was measured using the mouse IL-1β ELISA kit (Invitrogen) according to the manufacturer’s protocol.

### siRNA transfection

RAW264.7 cells were plated at 2×10^5^ cells per well in 24-well plates for 18 to 24 h at 37°C in a 5% CO2 humidified incubator before transfection. A total of 100 pM of siRNA was transfected using Lipofectamine 3000 reagent (Thermo Fisher Scientific). Forty-eight hours after transfection, the cells were infected with the indicated mycobacteria at an M.O.I. of 10 and harvested at 24 h. Acell SMART pool siRNAs targeting mouse cGAS (Mb21d1, 214763; #E-055528-00-0005) and STING (Tmem173, 72512; #E-055608-00-0005) and the corresponding Acell non-targeting siRNA (scramble) (#D-001910-10-05) from Dharmacon were used.

### Bacterial cell-to-cell spreading assay

J774A.1 cells were infected with recombinant EGFP-expressing bacteria called rEGFP_Mma-R (*M*. *massiliense* rough strain, A50594) and rEGFP_Msm (*M*. *smegmatis*) at an M.O.I. of 10. All EGFP-expressing recombinant bacteria were generated as described in a previous study [22, 56, 57]. After 2 h of infection, the infected cells were washed three times with PBS, and to remove extracellular mycobacteria, the cells were incubated with fresh culture medium containing 50 μg/ml amikacin. The supernatant was completely aspirated and washed with PBS three times, and then the trypsinized cells were collected. The cell-to-cell spreading was analysed by flow cytometry using a FACSCalibur (Becton-Dickinson, San Diego, CA).

### Enzyme-linked immunosorbent assay

The cells were infected in 6-, 12-, and 24-well plates. Post-infection cell culture supernatants were collected from infected cells and stored at -70°C. Paired antibodies and recombinant mouse IL-1β, TNF-α, IL-6, IL-10 (eBioscience), and IFN-β (BioLegend) were used as standards to determine cytokine concentrations according to the manufacturers’ instructions.

### Cytotoxicity assay

Cell cytotoxicity was determined using the CytoTox 96 Non-Radioactive Cytotoxicity Assay Kit, according to the manufacturer’s protocol (Promega). Lactate dehydrogenase (LDH) was released from cell with damaged membrane. Briefly, the cell supernatants were collected and incubated with the reaction mixture from the LDH assay Kit for 30 min, protected from light, at room temperature. After then, added the stop solution to each well of the plate and recorded absorbance at 490nm. The percent of cell cytotoxicity were calculated by using the formula: Percent cytotoxicity = Experimental LDH Release (OD_490_)/Maximum LDH Release (OD_490_) × 100%.

### Measurement of mitochondrial ROS detection

Cells were stained with the mitochondrial superoxide indicator MitoSOX (Invitrogen) as described in the manufacturer's protocols. Cells were loaded with 5 μM MitoSOX for 30 min and washed three times with PBS. MitoSOX fluorescence were measured using a FACSCalibur (Becton-Dickinson, San Diego, CA) and were gated and analysed using forward and side-scatter plots on 10,000 live events.

### RNA extraction and qRT-PCR

The cGAS-, STING- or scramble siRNA-transfected cells were processed by RNA extraction using TRIzol reagent (Invitrogen) according to the manufacturer's instructions. Equal amounts of total RNA (100 ng) were used to synthesize cDNA using the *Maxime* RT Premix kit (Intron). Gene expression was analysed by quantitative reverse transcription-PCR (RT-qPCR) with an ABI 7500 (Applied Biosystems, Grand Island, NY, USA) real-time machine using specific primers for STING, cGAS, IFI204, IFN-β, and IL-1β, and SYBR green PCR master mix (Applied Biosystems, Grand Island, NY, USA) as described previously [22].

### Detection of cytosolic mtDNA

J774A.1 cells were seeded onto 6-well plates and infected with various Mycobacteria strains at an M.O.I. of 10 for 24 h. Then, the infected cells were washed with PBS. Cytosolic cell fractions were isolated using the Qproteome Cell Compartment Kit (Qiagen) according to the manufacturer's protocol, and the procedures for preparing cellular fractions for detection of mtDNA have been previously described [22]. Using the PCI method, we obtained purified DNA from the fractionated supernatant. To detect cytosolic mycobacterial DNA, PCR was performed using the mycobacterial *hsp*65 gene [22]. Relative cytosolic mtDNA was measured by quantitative real-time PCR on an ABI 7500 (Applied Biosystems, Grand Island, NY, USA) using Fast SYBR Green Master Mix (Applied Biosystems, Grand Island, NY, USA), and specific primer sets that are listed in S1 Table. [58]. Three technical replicates were performed for each DNA sample, and the results were acquired as *C*_T_ values. The mtDNA amount from whole-cell extracts served as normalization for the mtDNA values obtained from the cytosolic cellular fractions.

### Measurements of oxidative DNA damage quantitation

A competitive ELISA for 8-OHdG was performed using a commercial 8-OHdG ELISA kit (OxiSelect Oxidative DNA Damage ELISA Kit 8-OHdG Quantitation, Cell Biolabs) according to the manufacturer’s instructions. The DNA samples were incubated at 95°C for 5 min and rapidly chilled on ice for single-stranded DNA. The DNA samples were digested with 5 units of nuclease P1 for 2 h at 37°C at a final concentration of 20 mM sodium acetate, pH 5.2 and treated with 5 units of alkaline phosphatase for 1 h at 37°C in a final concentration of 100 mM Tris, pH 7.5. The reaction mixture samples were centrifuged for 5 min at 6000×g, and the supernatant was used for the quantitation of oxidative DNA damage.

### Statistical analysis

All data were analysed using GraphPad Prism 5 and Microsoft Excel software. Band intensity of western blotting was also quantified by Image J software. All experiments were performed with two biological replicates and at least of two independent experiments. All data are shown as the means ± standard deviation (SD). Student’s *t*-test was used to compare two experimental groups. One-way or two-way ANOVA followed by Tukey’s multiple comparison test was performed for multiple-group comparisons. Asterisks indicate statistical significance as described in the corresponding figure legends (ns, no significant; *P < 0.05; **P < 0.01, and ***P < 0.001).

## Supporting information

S1 Fig Comparison of mtROS productions between various subspecies or genotypes of *M*. *abscessus* in mycobacteria-infected J774A.1 cells using MitoSOX. J774A.1 cells were infected with various subspecies or genotype strains at 10 M.O.I. for 24 h. Then, cells were pre-treated with rotenone (Rot; 5 μM) as a positive control (induction of ROS production) for 30 min. The infected cells were stained with MitoSOX and analysed by flow cytometry (FACSCalibur). Error bars represent the SD. Statistical significance was determined by ANOVA with Tukey's multiple comparison test.
(TIF)

S2 Fig Cytosolic mtDNA and oxidized DNA induced by MAB-R strains decreased in macrophages treated with cyclosporin A. (A) Cytosolic mtDNA was extracted from nuclear and cytosolic fractions of J774A.1 cells infected with strains of MAB-R, MAB-S, *M*. *smegmatis* (Msm) or *M*. *marinum* (Mmari) at an M.O.I. of 10 for 24 h. Measurement of cytosolic mtDNA expression by qRT-PCR was performed using the mitochondrial D-loop (D-loop-1, -2, and -3), CytB, ND4 and 16S primer sets. Normalization was performed as described in the materials and methods. (B) J774A.1 cells were pre-treated with cyclosporin A (CsA; 10 μM for 1 h) and infected with CFSE-labelled Mab-R or -S (green) strains at an M.O.I. of 10 for 24 h. Then, the infected cells were stained with anti-8-oxyhydrodioxy guanosine (8-OHdG) and DAPI (blue). All images were captured at 100× magnification. (C) J774A.1 cells were pre-treated with CsA and infected with strains of MAB-R, MAB-S or *M*. *smegmatis* (Msm) at an M.O.I. of 10 for 24 h. Cytosolic DNA was extracted from nuclear and cytosolic fractions of infected whole-cell lysates, and the levels of 8-OHdG were measured by ELISA. Error bars represent the SD. Statistical significance was determined by ANOVA with Tukey's multiple comparison test (A) two-tailed Student’s *t*-test (C).
(TIF)

S3 Fig Increased IFN-β and IL-1β production induced by MAB-R was mtROS-dependent. (A) J774A.1 cells were infected with various subspecies or genotype at 10 M.O.I for 24 h. Supernatants were collected from infected cells, and IL-1β levels were analysed by ELISA. (B) J774A.1 cells were pre-treated with mito-TEMPO (100 μM) or CsA (10 μM for 1 h) and infected with strains of MAB-R, MAB-S or *M*. *smegmatis* (Msm) at an M.O.I. of 10 for 24 h. Then, the infected cells were pre-treated with rotenone (5 μM) for 30 min as a positive control (induction of ROS production) and stained with MitoSOX and analysed by flow cytometry (FACSCalibur). (C) Supernatants from the infected cells in the presence of CsA were collected, and IL-1β and IFN-β cytokine levels were analysed by ELISA. (D) J774A.1 cells were pre-treated with mito-TEMPO or CsA and infected with strains of MAB-R, MAB-S or *M*. *smegmatis* (Msm) at an M.O.I. of 10 for 24 h. Supernatants from the infected cells were collected, and TNFα, IL-6 and IL-10 cytokine levels were analysed by ELISA. Error bars represent the SD. Statistical significance was determined by ANOVA with Tukey's multiple comparison test (A) two-tailed Student’s *t*-test (B-D).
(TIF)

S4 Fig mito-TEMPO treatment inhibited MAB-R escape into the cytosol in infected macrophages. J774A.1 cells were pre-treated with mito-TEMPO (100 μM) and infected with CFSE-labelled (green) Mab-R at an M.O.I. of 10 for 4 or 24 h. Then, the infected cells were stained with DAPI (blue). All images were captured at 100× magnification. The graph shows the result of intracellular bacterial numbers in each phagosome by randomly counting in 15 selected phagosomes in MAB-R-infected cells. Error bars represent the SD. Statistical significance was determined by two-tailed Student’s *t*-test.
(TIF)

S5 Fig MAB-R replication in J774A.1 cell phagosomes was inhibited by mito-TEMPO treatment. Untreated or mito-TEMPO (100 μM) pre-treated J774A.1 cells were infected with Mab-R at an M.O.I. of 10 for different times (2, 4, 8, 14 and 24 h). The infected cells were analysed by AFB staining and observed under a microscope at 100× magnification. The black arrows indicate Mab-R (red-stained bacilli) in methylene blue-stained J774A.1 cells.
(TIF)

S6 Fig Western blot analysis of cytosol, membrane and nuclear fractions from mycobacteria-infected J774A.1 cells. J774A.1 cells were lysed and the cytosolic, membrane and nuclear fractions were separated (see "Materials and Methods"). Then, 100 μg of protein extracts were separated and analysed by Western blotting. GAPDH was used as a cytosolic marker, and LAMP-1 was used as a phagosomal marker. The nuclear marker (Lamin B1) was detectable in nuclear extracts but not in the cytosol and membrane. MAB-R [Mma-R (*M*. *massiliense*, rough strain) and Mab-R (*M*. *abscessus*, rough strain)]; MAB-S [Mma-S (*M*. *massiliense*, smooth strain) and Mab-S (*M*. *abscessus*, smooth strain)]; Msm, *M*. *smegmatis*; TLS, J774A.1 total lysate.
(TIF)

S7 Fig Inhibition of mtROS reduced intracellular survival of MAB-R strains via inhibiting cell-to-cell spreading. (A-B) J774A.1 cells pre-treated with CsA (10 μM for 1h) and infected with strains of MAB-R, MAB-S or *M*. *smegmatis* (Msm) at 10 M.O.I for 24 h. Infected cell lysates were serially diluted and plated onto 7H10 agar plates for CFU assays (A) and supernatants were used for lactate dehydrogenase (LDH) assays (B). (C) J774A.1 cells were infected with various subspecies or genotype at an M.O.I. of 10 for 24 h. Infected cell lysates were serially diluted and plated onto 7H10 agar plates for CFU assays. (D) J774A.1 cells were pre-treated with mito-TEMPO (100 μM) and infected with luciferase-expressing recombinant mycobacterial strains, rLuci_Mma-R and rLuci_Msm (*M*. *smegmatis*) an M.O.I. of 10 for different times (2, 6 and 24 h). Infected cells were lysed with reporter lysis buffer (Promega) for 30 min at RT and mixed with firefly luciferin substrate (Promega), and luciferase intensities were measured by an illuminometer (TECAN). Error bars represent the SD. Statistical significance was determined by ANOVA with Tukey's multiple comparison test (C, left panel) two-tailed Student’s *t*-test (A-D).
(TIF)

S1 Table Primer sequences used in PCR. (XLSX)
