# Supplementary figures and images for: Mycobacterium abscessus infection leads to enhanced production of type 1 interferon and NLRP3 inflammasome activation in murine macrophages via mitochondrial oxidative stress
Source: PLoS Pathog. 2020 Mar 25;16(3):e1008294. doi: 10.1371/journal.ppat.1008294 (PMC7094820; doi:10.1371/journal.ppat.1008294)

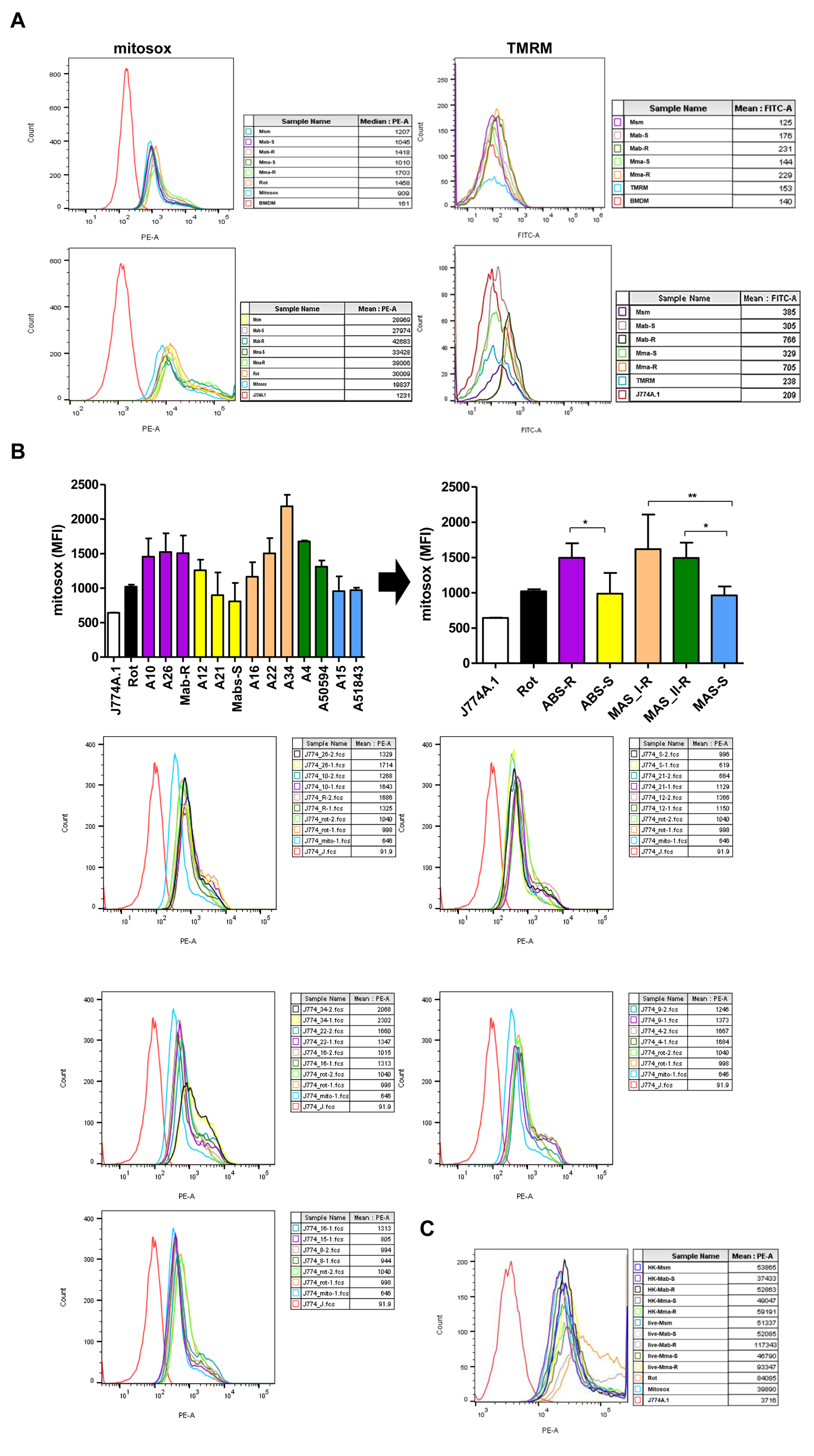

Supplement: S1 Fig — J774A.1 cells were infected with various subspecies or genotype strains at 10 M.O.I. for 24 h. Then, cells were pre-treated with rotenone (Rot; 5 μM) as a positive control (induction of ROS production) for 30 min. The infected cells were stained with MitoSOX and analysed by flow cytometry (FACSCalibur). Error bars represent the SD. Statistical significance was determined by ANOVA with Tukey's multiple comparison test. (TIF) [file ppat.1008294.s001.tif]

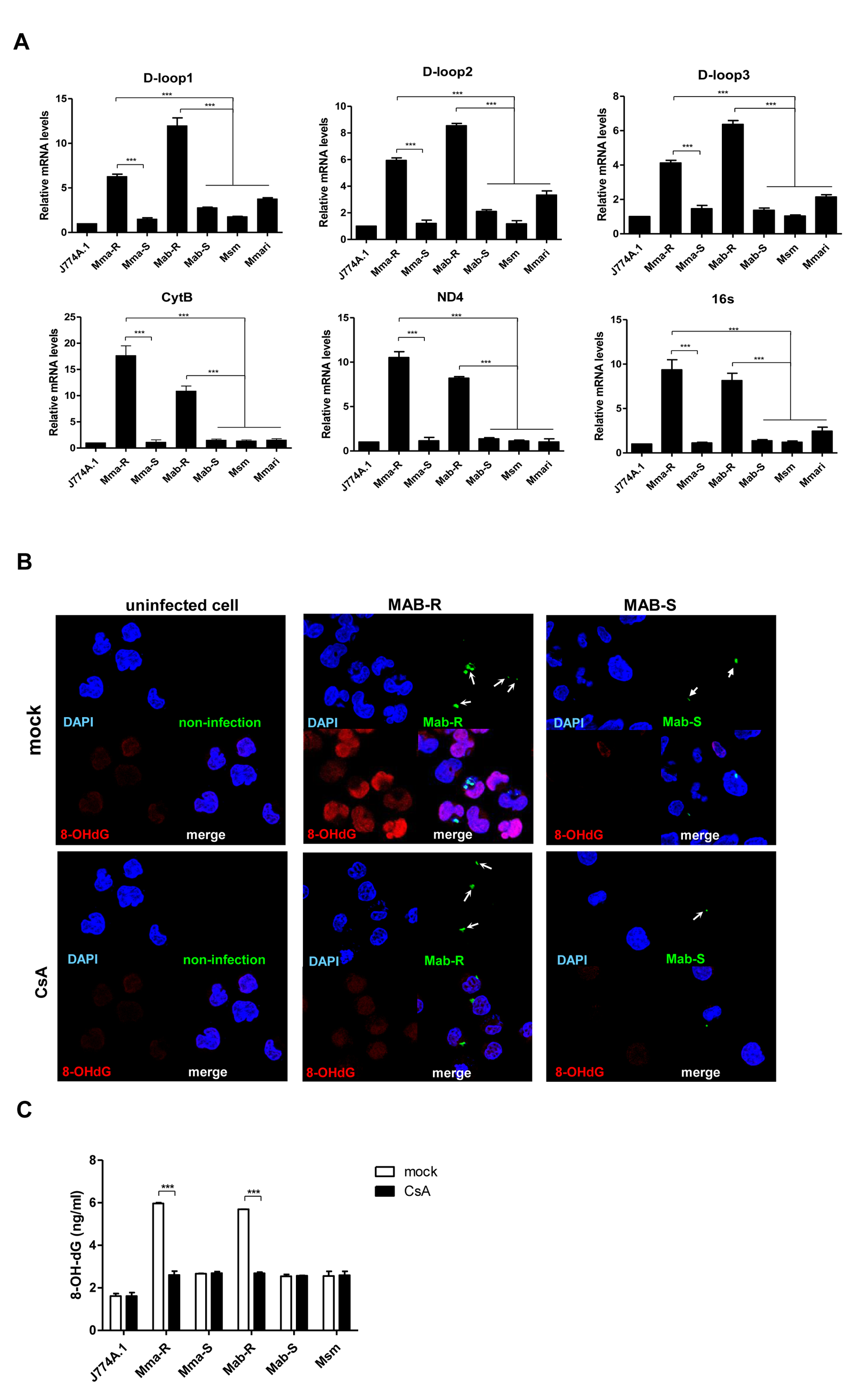

Supplement: S2 Fig — (A) Cytosolic mtDNA was extracted from nuclear and cytosolic fractions of J774A.1 cells infected with strains of MAB-R, MAB-S, M. smegmatis (Msm) or M. marinum (Mmari) at an M.O.I. of 10 for 24 h. Measurement of cytosolic mtDNA expression by qRT-PCR was performed using the mitochondrial D-loop (D-loop-1, -2, and -3), CytB, ND4 and 16S primer sets. Normalization was performed as described in the materials and methods. (B) J774A.1 cells were pre-treated with cyclosporin A (CsA; 10 μM for 1 h) and infected with CFSE-labelled Mab-R or -S (green) strains at an M.O.I. of 10 for 24 h. Then, the infected cells were stained with anti-8-oxyhydrodioxy guanosine (8-OHdG) and DAPI (blue). All images were captured at 100× magnification. (C) J774A.1 cells were pre-treated with CsA and infected with strains of MAB-R, MAB-S or M. smegmatis (Msm) at an M.O.I. of 10 for 24 h. Cytosolic DNA was extracted from nuclear and cytosolic fractions of infected whole-cell lysates, and the levels of 8-OHdG were measured by ELISA. Error bars represent the SD. Statistical significance was determined by ANOVA with Tukey's multiple comparison test (A) two-tailed Student’s t-test (C). (TIF) [file ppat.1008294.s002.tif]

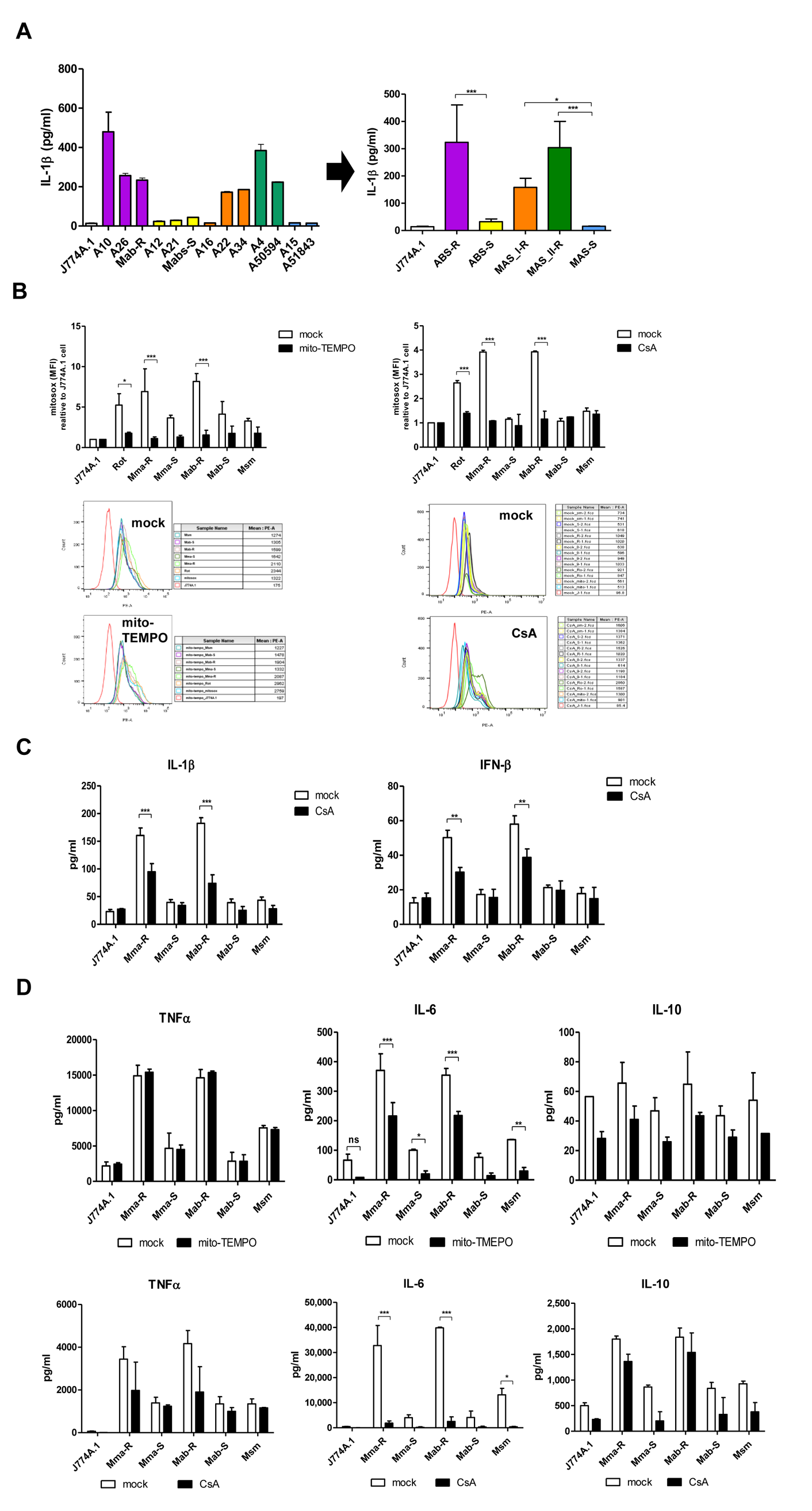

Supplement: S3 Fig — (A) J774A.1 cells were infected with various subspecies or genotype at 10 M.O.I for 24 h. Supernatants were collected from infected cells, and IL-1β levels were analysed by ELISA. (B) J774A.1 cells were pre-treated with mito-TEMPO (100 μM) or CsA (10 μM for 1 h) and infected with strains of MAB-R, MAB-S or M. smegmatis (Msm) at an M.O.I. of 10 for 24 h. Then, the infected cells were pre-treated with rotenone (5 μM) for 30 min as a positive control (induction of ROS production) and stained with MitoSOX and analysed by flow cytometry (FACSCalibur). (C) Supernatants from the infected cells in the presence of CsA were collected, and IL-1β and IFN-β cytokine levels were analysed by ELISA. (D) J774A.1 cells were pre-treated with mito-TEMPO or CsA and infected with strains of MAB-R, MAB-S or M. smegmatis (Msm) at an M.O.I. of 10 for 24 h. Supernatants from the infected cells were collected, and TNFα, IL-6 and IL-10 cytokine levels were analysed by ELISA. Error bars represent the SD. Statistical significance was determined by ANOVA with Tukey's multiple comparison test (A) two-tailed Student’s t-test (B-D). (TIF) [file ppat.1008294.s003.tif]

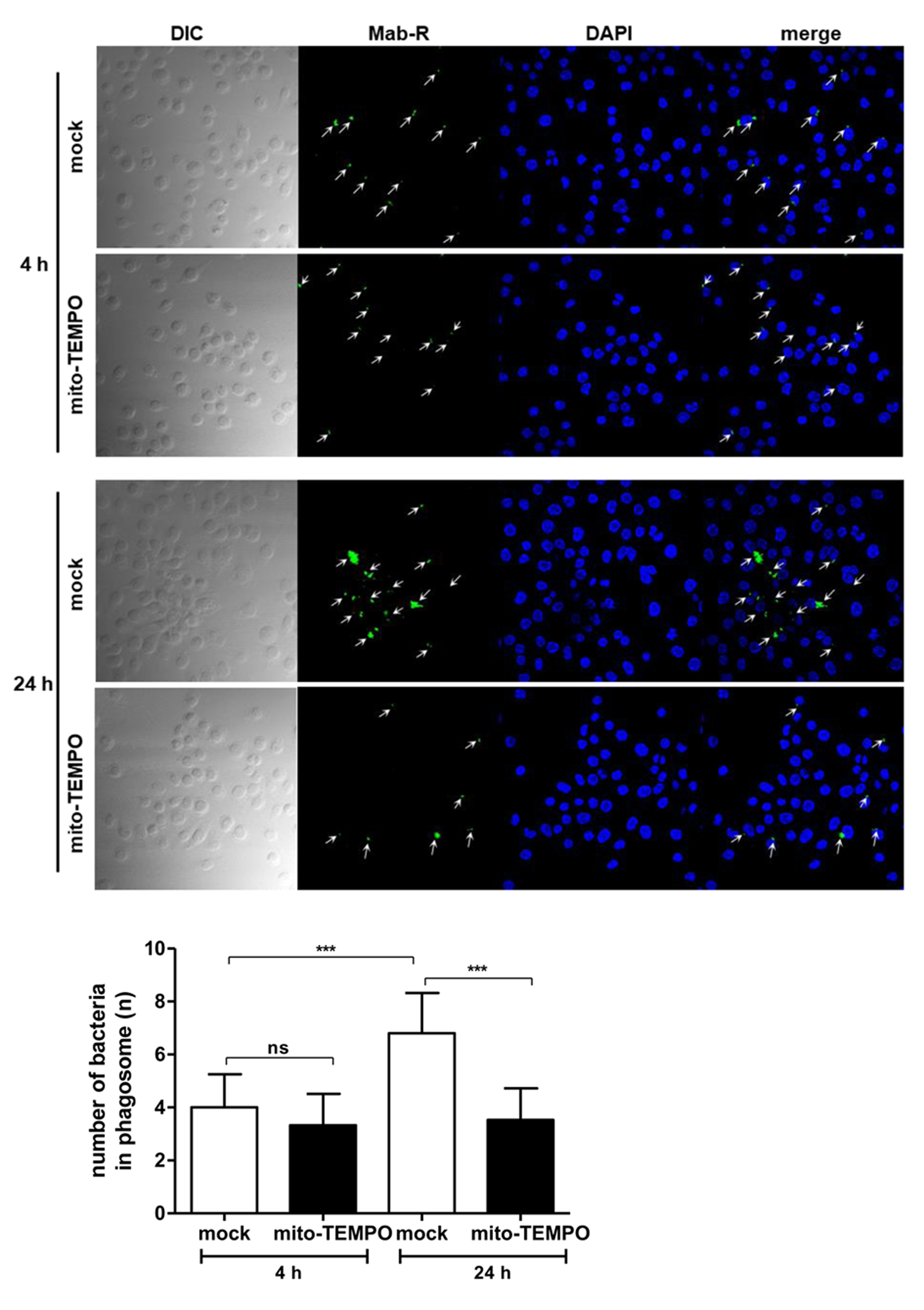

Supplement: S4 Fig — J774A.1 cells were pre-treated with mito-TEMPO (100 μM) and infected with CFSE-labelled (green) Mab-R at an M.O.I. of 10 for 4 or 24 h. Then, the infected cells were stained with DAPI (blue). All images were captured at 100× magnification. The graph shows the result of intracellular bacterial numbers in each phagosome by randomly counting in 15 selected phagosomes in MAB-R-infected cells. Error bars represent the SD. Statistical significance was determined by two-tailed Student’s t-test. (TIF) [file ppat.1008294.s004.tif]

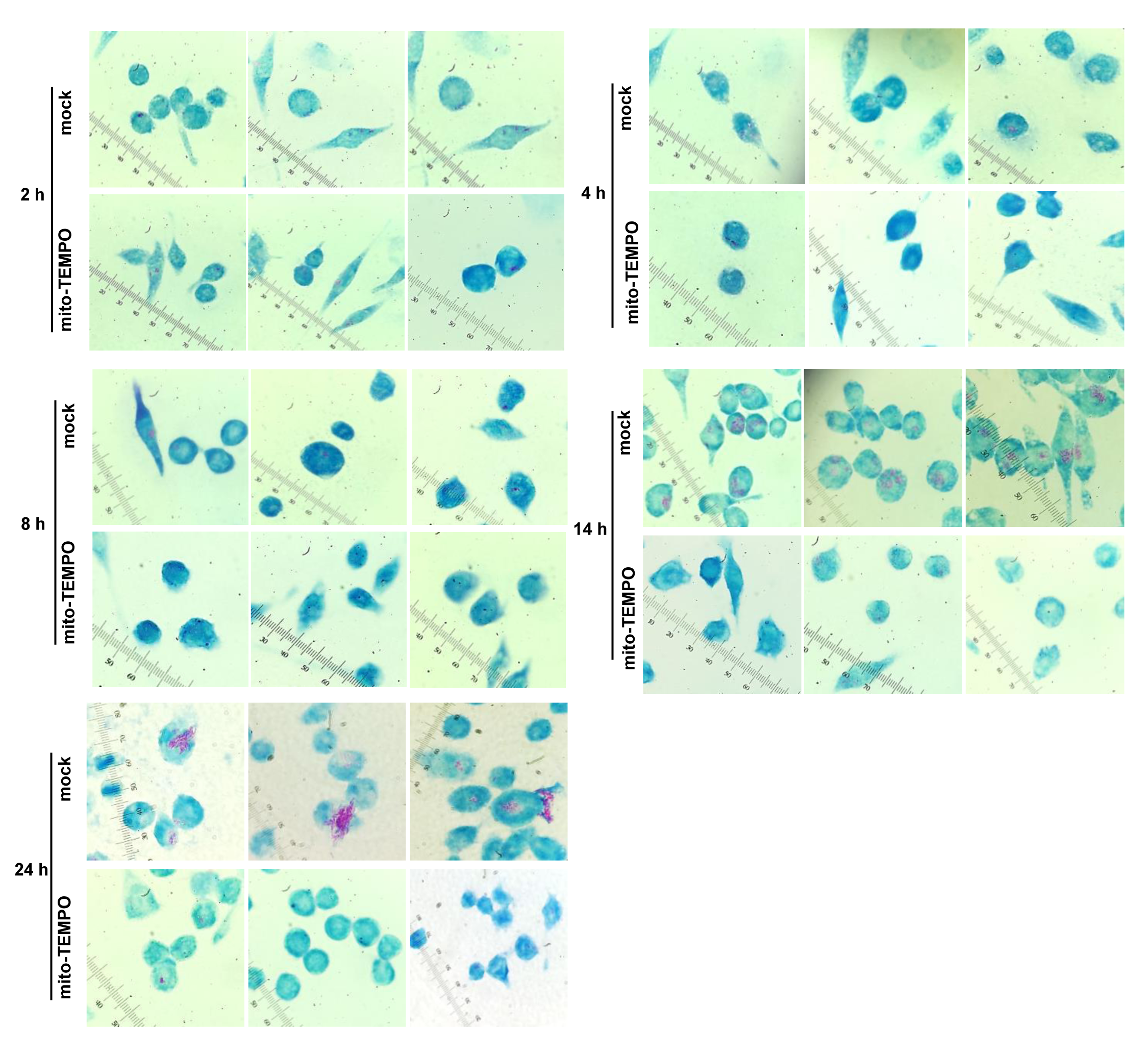

Supplement: S5 Fig — Untreated or mito-TEMPO (100 μM) pre-treated J774A.1 cells were infected with Mab-R at an M.O.I. of 10 for different times (2, 4, 8, 14 and 24 h). The infected cells were analysed by AFB staining and observed under a microscope at 100× magnification. The black arrows indicate Mab-R (red-stained bacilli) in methylene blue-stained J774A.1 cells. (TIF) [file ppat.1008294.s005.tif]

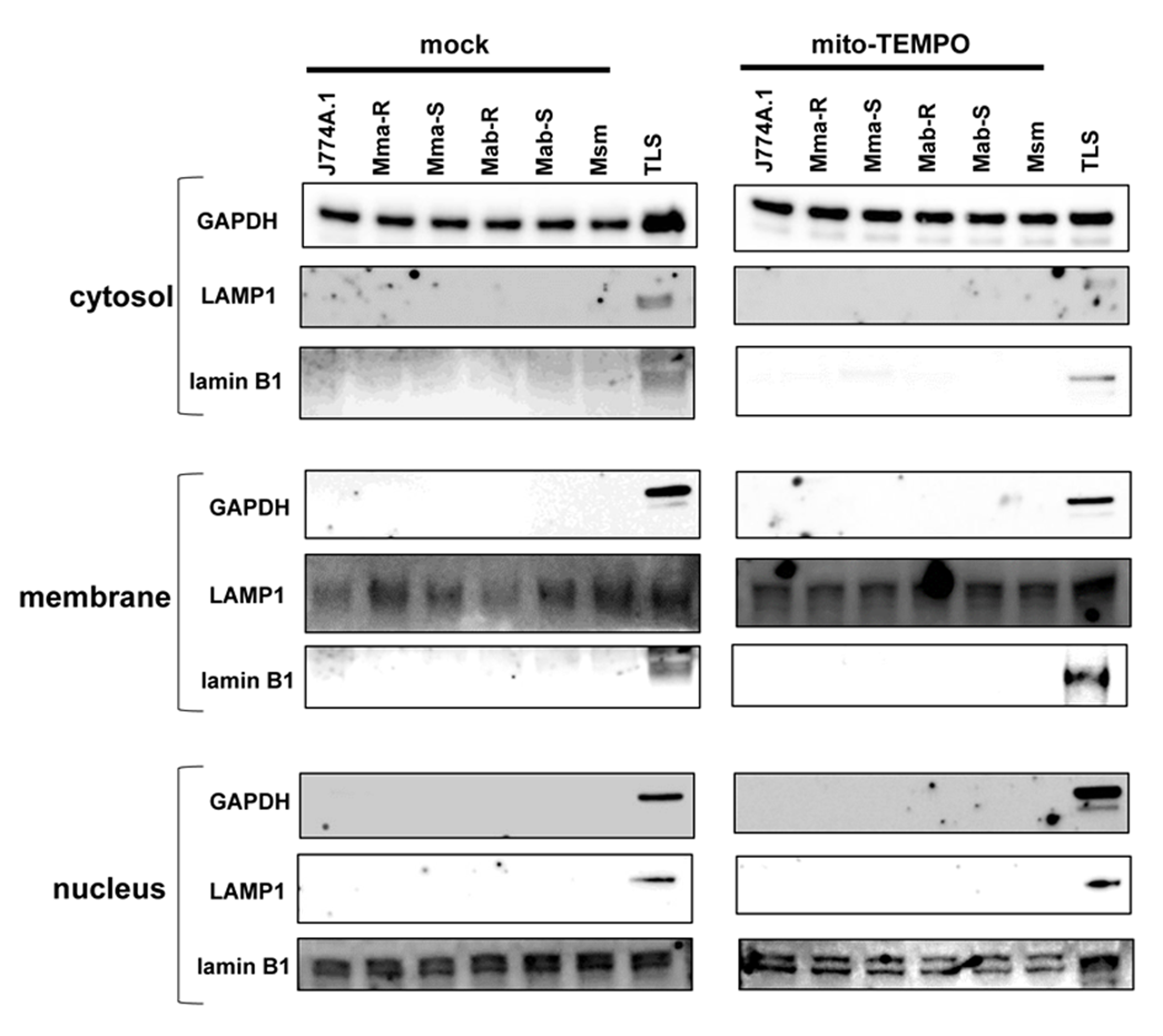

Supplement: S6 Fig — J774A.1 cells were lysed and the cytosolic, membrane and nuclear fractions were separated (see "Materials and Methods"). Then, 100 μg of protein extracts were separated and analysed by Western blotting. GAPDH was used as a cytosolic marker, and LAMP-1 was used as a phagosomal marker. The nuclear marker (Lamin B1) was detectable in nuclear extracts but not in the cytosol and membrane. MAB-R [Mma-R (M. massiliense, rough strain) and Mab-R (M. abscessus, rough strain)]; MAB-S [Mma-S (M. massiliense, smooth strain) and Mab-S (M. abscessus, smooth strain)]; Msm, M. smegmatis; TLS, J774A.1 total lysate. (TIF) [file ppat.1008294.s006.tif]

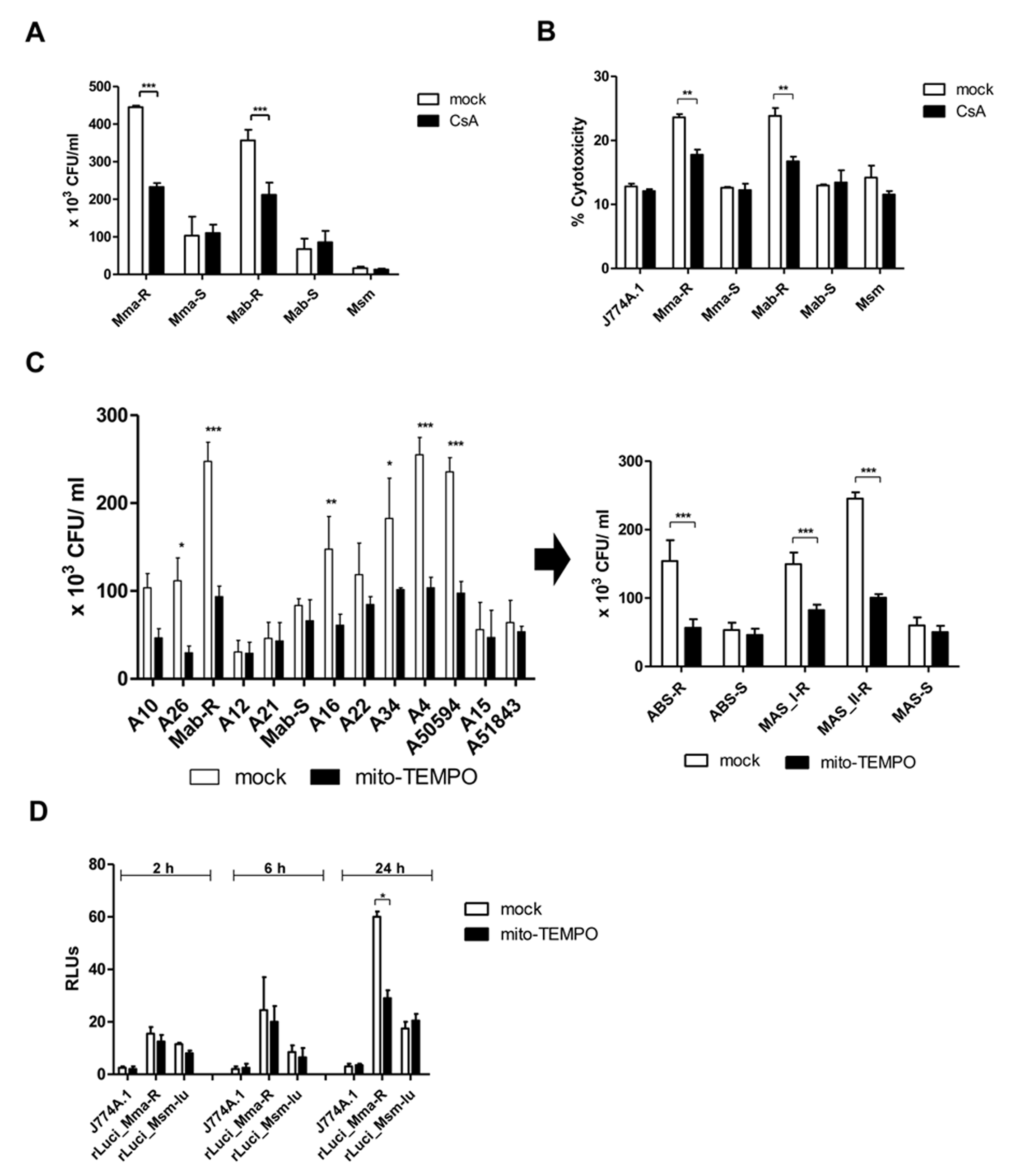

Supplement: S7 Fig — (A-B) J774A.1 cells pre-treated with CsA (10 μM for 1h) and infected with strains of MAB-R, MAB-S or M. smegmatis (Msm) at 10 M.O.I for 24 h. Infected cell lysates were serially diluted and plated onto 7H10 agar plates for CFU assays (A) and supernatants were used for lactate dehydrogenase (LDH) assays (B). (C) J774A.1 cells were infected with various subspecies or genotype at an M.O.I. of 10 for 24 h. Infected cell lysates were serially diluted and plated onto 7H10 agar plates for CFU assays. (D) J774A.1 cells were pre-treated with mito-TEMPO (100 μM) and infected with luciferase-expressing recombinant mycobacterial strains, rLuci_Mma-R and rLuci_Msm (M. smegmatis) an M.O.I. of 10 for different times (2, 6 and 24 h). Infected cells were lysed with reporter lysis buffer (Promega) for 30 min at RT and mixed with firefly luciferin substrate (Promega), and luciferase intensities were measured by an illuminometer (TECAN). Error bars represent the SD. Statistical significance was determined by ANOVA with Tukey's multiple comparison test (C, left panel) two-tailed Student’s t-test (A-D). (TIF) [file ppat.1008294.s007.tif]
