# Supplementary material for: Mycobacterium abscessus infection leads to enhanced production of type 1 interferon and NLRP3 inflammasome activation in murine macrophages via mitochondrial oxidative stress
Source: PLoS Pathog. 2020 Mar 25;16(3):e1008294. doi: 10.1371/journal.ppat.1008294 (PMC7094820; doi:10.1371/journal.ppat.1008294)
Supplement: Attachment — Submitted filename: Reply to editors and reviewers (BR Kim).docx [file ppat.1008294.s009.docx]

[EXSCINDED]Dear Prof. Kim:

Reply to Editor’s comments

1. Specifically, these are the major criticisms that need to be addressed: experiments that specifically and directly address the role of NLRP3 and IFNAR in the response to M. abscessus (use of genetic KO strategies or blocking antibodies), additional assays to assess oxidative stress pathways, and addition of CFU assays for key experiments.

🡪 In this study, to check the role of NLRP3 and IFNAR in MAB-R infection, we tested cytokine (Fig 7A and 7B) and CFU assay (Fig 7C) using interleukin-1β receptor (IL-1R1) blocking antibodies or IFNAR1 KO mice.

2. In addition, the reliance of the major conclusions on 2 strains of M. abscessus raises the risk of falsely attributing the findings to the R vs S phenotype rather than some other difference. Analysis of additional strains or further analysis of the studied strains would be needed to at least partially understand the role of the strain choice when interpreting the major conclusions.

🡪 To demonstrate our hypothesis, various genotypes of MAB were used in this study. The mycobacterial strains representatively used in this study are as follows: 2 clinical isolates of *Mycobacterium abscessus* [(ATCC 19977 ^T^ rough (Mab-R) and smooth type (Mab-S)], *Mycobacterium smegmatis* ATCC 19420^T^ and *Mycobacterium marinum* JCM 17638^T^ and 2 clinical isolates of *Mycobacterium abscessus* subsp. *massiliense* type I smooth (Mma-S), type II-rough (Mma-R). Other various genotype mycobacteria, *M. abscessus* rough strains (ABS-R); A10 and A26; *M. abscessus* smooth strains (ABS-S): A12 and A21; *M. massiliense* type I-rough strains (MAS_I-R): A16, A22 and A34; *M. massiliense* type II-rough strains (MAS_II-R): A4 and A50594; *M. massiliense* smooth strains (MAS-S): A15 and A51843, were used.

Reply to reviewer’s comments

I. Major Issues

1) Reviewer #1:
1. In fig. 2 and 4, the authors need to show that cytosolic mtDNA and oxidized mtDNA accumulation by additional methods such as immunostaining methods.

🡪 In the revised version, the new data of immune fluorescence (IF) staining were included as Fig 2B and S2B Fig.

2. The authors only used Mito-TEMPO to regulate mitochondrial oxidative stress and cytosolic oxidized mtDNA. It is not enough to support their demonstration and exclude the side effect of a single drug. The authors need to add other ROS scavengers and regulate mitochondrial oxidative stress with gene silencing.

🡪 As you recommended, we investigated whether other ROS reagents can regulate mtROS in the same with mito-TEMPO. We used the general ROS antagonist N-acetylcysteine (NAC), is known as an inhibitor of ROS. Our results showed that mito-TEMPO significantly could reduce the mitochondrial oxidative stress and cytosolic oxidized mtDNA, whereas NAC was not effective to regulate mtROS in MAB-R infection (below figure). NAC failed to reduce mitochondrial ROS (MitoSox) and oxidized mtDNA in MAB-R infected macrophages (below Fig A and B). Also, the levels of IL-1β secretion were decreased, whereas there were no significant differences in IFN-β production and cytotoxicity between NAC treatment and no treatment (below Fig C and D). Together, these results suggest that mitochondrial-targeted antioxidant (mito-TEMPO) was more effective than general ROS scavenger (NAC), highlighting the importance of a direct-mitochondria targeted ROS approach.

Similarly, recent studies have reported that mitochondrial-targeted antioxidants (mito-TEMPO) can be used as potential therapeutic candidates compared to NAC (Z Wang *et al*., The Role of Mitochondria-Derived Reactive Oxygen Species in Hyperthermia-Induced Platelet Apoptosis. PLoS ONE (2013) 8(9): e75044. and C McCarthy *et al*., Therapeutically targeting mitochondrial redox signalling alleviates endothelial dysfunction in preeclampsia Sci Rep. (2016) Sep 8;6:32683.)

3. Furthermore, the authors need to show the effect of cytosolic oxidized mtDNA accumulation specifically through regulating the release of mtDNA into cytosol by using inhibitors of mitochondria pore opening such as cyclosporine A.

🡪 In the revised version, the new data of oxidized mtDNA accumulation in the presence of cyclosporine A were included. Our data showed in S2B Fig (8-OHdG confocal and 8-OHdG ELSIA), S3B Fig (mtROS), S3C and S3D Fig (the level of IL-1β, IFNβ, TNFα, IL-6, IL-10). Consistent with data from results of mito-TEMPO treated macrophage cells induced by MAB-R, a similar trends of results were also observed in cylocsporin A(CsA) treated J774A.1 cells infected with MAB-R strains.

4. There is no clue to discriminate whether bacterial replication within phagosomes were affected by inflammatory cytokines induced by oxidized mtDNA or regulated by mtROS directly after mito-TEMPO treatment.

🡪 In this study, our results showed that treatment of mito-TEMPO significantly inhibited bacterial replication in MAB-R infected macrophages after 24 h infection. However, there were no significant differences in bacteria number between mito-TEMPO treatment and no treatment 4 h after infection (S4 Fig) (page 8, line 192~197). Also We found that the treatment of mito-TEMPO led to enhanced co-localization with LAMP-1 24h after infection, but not 4h after infection (Fig 4A). However, it is not true in case without the treatment with mito-TEMPO. These suggest that mtROS induced by MAB-R infection led to their intracellular survival via phagosomal rupture in murine macrophage. Our TEM (Fig 4B) and AFB staining analysis (Fig 4C and S5 Fig) also showed a positive role of mtROS in bacteria active replication within phagosome in MAB-R infected macrophages, further supporting our above findings. (page 9, line 200~210)

🡪 Furthermore, as shown in below Figure, IL-1β production induced by MAB-R strains was released after 18 h. Therefore, we suggested that the bacterial replication of MAB-R strains within phagosomes was affected by mtROS induction, not inflammatory cytokines (IL-1β).

5. Also they didn’t show any effect of inflammasome activation on MAB infection. They need to investigate the effect of inflammasome activation or type I IFN production using IL-1 or IFN-I receptor blocking or depletion of signaling intermediate molecules.

🡪 In the revised version, as shown in Fig 7, IL-1β and IFN-I productions induced by MAB-R infection were examined in BMDMs from IFNR1 KO mice or in J774A.1 cells subject to interleukin-1 receptor (IL-1R1) blocking antibodies (Abs).

6. The authors need to verify that NLRP3 inflammasome or cGAS-sting pathway really recognize cytosolic oxidized mtDNA and activate inflammasome or type I IFN signaling pathway respectively in MAB infection by using gene silencing assay or KO mice when possible.

🡪 In the revised version, the new data of siRNA knockdown assay was included as a Fig 6. We described that IFN-I signaling found in MAB-R infected cells depends on cGAS-STING pathway and NLRP3 mediated IL-1β production found in MAB-R infected cells was independent of cGAS-STING pathway. (page 11, line 259~269)

7. The discussion need revision to reflect the importance of this finding.

🡪 As you recommended, we revised the previous manuscript and uploaded new version.

2) Reviewer #2:
8. I am not sure of the relationship between Type I interferons, IL-1b induction, mitochondrial stress, and disease pathogenesis. I understand that inhibition of mitochondrial stress appears to be fundamentally beneficial given that replication was diminished in the setting of mitochondrial stress inhibition, but is this phenotype dependent on T1 interferon or would it be worse without IL-1b? More discussion of this somewhat, but not entirely, surprising phenotype would help me place stress, inflammasome, and type I interferon expression in better context. If possible, repeating the experiment in figure 7 with il-1r and ifnar deficient bmdm would be compelling as well.

🡪 As you recommended, we added the schematic figure including IL-1R1 and IFNAR1 KO (Fig 8).

9. Although I appreciate the western blot data, I would like to see some sort of aggregate quantitation with statistical analysis for data that demonstrates more reliability of this data.

🡪 As you recommended, we added bar graphs analyzed by Image J program (Fig 3D and 6A).

II. Minor Issues

1) Reviewer #1:
1. The authors mentioned that IL-6 production was not significantly different between live and HK MAB (p7, l161). However, mito-TEMPO significantly inhibited the production of IL-6 comparable to IFN- or IL-1 in Fig 4. What makes this discrepancy?

🡪 As you recommended, we rewrote the revised manuscript as follows, “Our data showed that treatment with mito-TEMPO or CsA inhibited IL-1β and IFN-β production in MAB-R-infected cells (Fig 3C and S3C Fig). A similar trend was also observed in IL-6 production in infected J774A.1 cells, suggesting that IFN-I, IL-1β and IL-6 production in cells infected by MAB-R strains depends on mtROS induction (S3D Fig). However, treatment with mito-TEMPO or CsA did not affect the production of TNF-α and IL-10, suggesting that their production does not depend on mtROS induction (S3D Fig).” (page 7~8, line 167~172)

2. The authors need to add cytotoxicity assay in Fig 3

🡪 As you recommended, we added cytotoxicity assay in MAB-R and –S strains infected macrophage cells in the presence of mito-TEMPO or cyclosporine A in the result section (Fig 5C and S7B Fig). (page 11, line 246~249)

🡪 Also, our previous study examined that cytotoxicity of BMDMs infected with MAB-R and –S strains and measured by lactate dehydrogenase (LDH) assay for 4 and 24 h. There were no significant differences in cytotoxicity between MAB-S and MAB-R infected cells in early infection time (4 h), but MAB-R strains showed more cytotoxicity in 24 h than other mycobacterial strains (MAB-S, M. smegmatis or BCG). Also we found that only live MAB-R strains led to significantly greater cell cytotoxicity than HK bacteria, although in other mycobacterial strains (MAB-S, M. smegmatis or BCG), no significant difference in cell death-inducing capacity between live and HK bacteria (BR Kim *et al*., Phagosome Escape of Rough Mycobacterium abscessus Strains in Murine Macrophage via Phagosomal. Front Immunol. 2019; 10: 125.).

3. The authors need to add CFU assay in Fig 6.

🡪 As you recommended, we added CFU assay data in the Fig 5B and S7C. To verify the intracellular survival in MAB-R infection in the presence of mito-TEMPO or cyclosporine A (CsA), colony forming units (CFUs) were enumerated from MAB-R or -S infected cell. We found that treatment with mito-TEMPO or CsA reduced the CFUs of MAB-R, but not MAB-S in infected J774A.1 cells (Fig 5B). Also, we checked that CFUs in various clinical genotype, MAB-R strains also reduced in the infected J774A.1 cells with mito-TEMPO treatment (S7C Fig).

2) Reviewer #2:
1. Line 124: Please add explanation for these abbreviations at the first time they appear in the text. Definitions for abbreviations were placed at the 2nd time they appeard in line 140.

🡪 We removed the duplicates abbreviations in the revised manuscript.
Introduction and throughout: There is mention of "proving" various hypotheses. Although this is a bit pedantic, I'm not sure that we as biologists are proving anything. Please reword

🡪 As you recommended, we removed the word “proving” in the revised manuscript.

2. Line 189. The text describes this data in Figure 4 incorrectly. It states "We found that treatment with mito-TEMPO lead to complete inhibition…" Data in Figure 4 demonstrates that IFNb is not at all completely inhibited, and likely IFNb remains statistically higher in Mab-R compared with Mab-S isolates after inhibition of mito stress. Please pay more attention to the description of your data

🡪 As you recommended, we described a sentence in the revised manuscript as follows, "First, we determined whether the mtROS induced by MAB-R strains was decreased by mito-TEMPO or CsA (S3B Fig). Our data showed that treatment with mito-TEMPO or CsA inhibited IL-1β and IFN-β production in MAB-R-infected cells (Fig 3C and S3C Fig).” (Page 7, line 165~168)

3. Line 228. The description of the experimental results in Figure 6C is extremely unclear and makes interpretation of the figure impossible. Please reword and provide clarification of the experiment and the results here.

🡪 As you recommended, we added the sentences in manuscript as follows. “To further determine whether mtROS leads to the escape of MAB-R strains into the cytosol during MAB-R infection, we conducted PCR analysis of the cytosolic fraction of MAB-R-infected macrophages. These results also indicated that mito-TEMPO treatment inhibited MAB-R escape into the cytosol in infected macrophages (Fig 4D and S6 Fig).” (page 9, line 211~214)

🡪 our previous study found that the phagosomal escape of MAB-R strains during infection analyzed by mycobacterial DNA detection in cytosol using mycobacterial hsp65 gene (BR Kim *et al*., Phagosome Escape of Rough Mycobacterium abscessus Strains in Murine Macrophage via Phagosomal. Front Immunol. 2019; 10: 125.). In order to confirm the effect of mtROS on cytosol escape of MAB-R strains, the presence of bacterial DNA in the cytosol of MAB-R infected J774A.1 cells was confirmed by PCR under mtROS inhibited condition (Fig 4D). 24 h after infection, MAB-R DNA was detected in the cytosol of infected cells, but not in the mito-TEMPO treated cells. At 4 h post infection, mycobacterial DNA was not detected in the cytosol of infected cells regardless of mito-TEMPO treatment (Fig 4D). In addition, the cellular fraction was confirmed by western blot for each cellular marker [GAPDH (cytosol), LAMP-1 (membrane) and Lamin B1(nucleus)] (S6 Fig).

4. Line 265. The Figure Legends are located before the discussion. Please place them at the end of the manuscript in their proper location.

🡪 We replaced the Figure Legends location as you recommended.

5. Line 387. I am confused by what is being described by a "vita-PAMP." Please either define further or eliminate this noncanonical term from the manuscript

🡪 We rewrote the revised manuscript as you recommended.
